# The association between genetic variants at 3’-UTR and 5’-URR of HLA-G gene and the clinical outcomes of patients with leukemia receiving hematopoietic stem cell transplantation

**DOI:** 10.3389/fimmu.2023.1093514

**Published:** 2023-02-23

**Authors:** Ding-Ping Chen, Po-Nan Wang, Ai-Ling Hour, Wei-Tzu Lin, Fang-Ping Hsu, Wei-Ting Wang, Ching-Ping Tseng

**Affiliations:** ^1^ Department of Laboratory Medicine, Linkou Chang Gung Memorial Hospital, Taoyuan, Taiwan; ^2^ Department of Medical Biotechnology and Laboratory Science, College of Medicine, Chang Gung University, Taoyuan, Taiwan; ^3^ Division of Hematology-Oncology, Department of Internal Medicine, Linkou Chang Gung Memorial Hospital, Taoyuan, Taiwan; ^4^ Department of Life Science, Fu Jen Catholic University, New Taipei City, Taiwan; ^5^ Graduate Institute of Biomedical Sciences, College of Medicine, Chang Gung University, Taoyuan, Taiwan

**Keywords:** hematopoietic stem cell transplantation (HSCT), single nucleotide polymorphism (SNP), human leukocyte antigen-G (HLA-G), graft versus host disease (GVHD), haplotype

## Abstract

In addition to the classical human leukocyte antigen (HLA) genes, the outcomes of post-hematopoietic stem cell transplantation (HSCT) are associated with human leukocyte antigen (HLA)-related genes and non-HLA genes involved in immune regulation. HLA-G gene plays an important role in immune tolerance, assisting immune escape of tumor cells, and decrease of transplant rejection. In this study, we explored the association of genetic variants at the 3’-untranslated region (3’-UTR) and 5’-upstream regulatory region (5’-URR) of HLA-G gene with the adverse outcomes of patients with leukemia receiving HSCT. The genomic DNAs of 164 patients who had acute leukemia and received HSCT were collected for analysis. Nine single nucleotide polymorphisms (SNPs) and six haplotypes in the 3’-UTR and 27 SNPs and 6 haplotypes in the 5’-URR were selected to investigate their relationship with the development of adverse outcomes for patients receiving HSCT, including mortality, relapse, and graft-versus-host disease. Our results revealed that two SNPs (rs371194629 and rs9380142) and one haplotype (UTR-3) located in the 3’-UTR and two SNPs (rs3823321 and rs1736934) and one haplotype (G0104a) located in the 5’-URR of HLA-G were associated with the occurrence of chronic GVHD or development of any forms of GVHD. No SNP was found to associate with the occurrence of mortality and relapse for patients receiving HSCT. These SNPs and haplotypes may play important roles in regulating immune tolerance of allografts post-HSCT that can be used to predict the risk of poor outcomes after receiving HSCT and giving preventive treatment to patients on time.

## Introduction

Hematopoietic stem cell transplantation (HSCT) is a treatment of patients with blood disorders or cancer (1). Autologous or allogeneic hematopoietic stem cells are delivered into patients to achieve the purpose of immunotherapy or support patients to receive high-dose radiotherapy or chemotherapy. In allogeneic transplantation, human leukocyte antigen (HLA)-matched stem cells must be used to avoid transplantation rejection. However, there are still cases of transplant failure even using HLA-matched stem cells (2, 3). It is speculated that other factors in addition to HLA may contribute to the occurrence of adverse outcomes such as disease relapse, mortality, and graft-versus-host disease (GVHD) for patients post-HSCT (4, 5).

HLA-A, HLA-B, and HLA-C are the human major histocompatibility complex (MHC) class I proteins which are well documented to associate with the outcomes of HSCT (6). An additional non-classical MHC class Ib gene HLA-G is located on human chromosome 6p21.3, adjacent to the HLA-A locus. HLA-G gene has 8 exons separated by 7 introns. Alternative splicing of HLA-G mRNA selectively produces 7 mature mRNA subtypes, encoding 7 protein isoforms which include 4 membrane-bound proteins (HLA-G1, G2, G3, and G4) and 3 soluble proteins (HLA-G5, G6, and G7) (7). HLA-G1 and HLA-G5 combine with beta-2-microglobulin together to form HLA-G complexes which are the main immunomodulatory subtype with biological functions in the HLA-G family (8, 9). HLA-G has been shown recently to play important roles in immune tolerance of organ transplants (10), immune escape of tumor cells and viruses (11), and fetus-maternal tolerance leading to protection of fetus from attack by the maternal immune system (7, 12). The study by Boukouaci et al. further demonstrated that HLA-G is induced *in vivo* during allogeneic transplantation leading to immune tolerance and reducing the risk of transplant rejection and GVHD (13). In *ex vivo* study, HLA-G expression on the surface of CD4^+^ T cells inhibits the proliferation of allogeneic-specific T cells when mixing with the allogenic lymphocytes (14). These studies indicate that HLA-G is highly related to immune reaction and tolerance.

The 3’-untranslated region (3’-UTR) and the 5’-upstream regulatory region (5’-URR) of HLA-G gene are highly polymorphic. Genetic variations in these regions are known to regulate the levels of HLA-G gene expression and post-transcriptional mRNA stability (15). In addition to the genetic variants of single nucleotide polymorphisms (SNPs) and insertion/deletion (INDEL), 6 haplotypes in the 3’-UTR and 6 haplotypes in the 5’-URR of the HLA-G gene have been defined (16). Genetic polymorphisms and haplotypes of HLA-G have been reported to associate with the risk and prognosis of cancer, organ transplantation, viral and parasitic infections, pregnancy complications, the clinical outcome of patients with beta-thalassemia receiving HSCT, and the susceptibility of autoimmune diseases (17–21). Nevertheless, whether there is an association between HLA-G genetic polymorphism and haplotypes with the risk of adverse outcomes for patients with leukemia receiving HSCT is still not clear.

The aim of this study was to investigate the effects of genotype and haplotypes in the 3’-UTR and 5’-URR of HLA-G gene on the mortality, the risk of disease relapse, and the development of GVHD for patients with acute myeloid leukemia (AML) and acute lymphoblastic leukemia (ALL) after receiving HSCT. The significance of the findings in this study was discussed.

## Methods and materials

### Patients and HLA typing

The study has been approved by the Institutional Review Board (IRB) of Chang Gung Memorial Hospital (CGMH) and was performed according to the ethical regulations and requirements. The approved ID was 201304949B0 and 202100302B0. Because HLA-G was mainly expressed in the saliva, peritoneal fluid, plasma, thymus, semen, cerebrospinal fluid but not in the immune cells (7), and only the HLA haplotypes of the recipients but not donors were associated with the outcomes after HSCT (18, 22), the analysis focused on the genetic components of the recipients. The inclusion criteria of participants were male and female adults (over 20 years old) who suffered from AML or ALL and underwent HSCT. Exclude the patients who had other hematological malignancies. A total of 164 patients receiving HSCT was enrolled in this study, in which 106 patients were diagnosed as AML and 58 patients were ALL. The clinical characteristics of the 164 patients are shown in **Table 1**. All recipients of unrelated HSCT signed informed consents and had fully matched HLA with the donors as revealed by using the SeCore kit (Thermo Fisher, Waltham, MA) for high-resolution HLA typing. The ambiguous alleles of the SeCore typing were resolved by using the MicroSSP Allele Specific Tying Tray with sequence-specific primers (Thermo Fisher, Waltham, MA).

**Table 1:** Clinical characteristics of patients who enrolled in the study.

Clinical parameters	Number of patients	percentage
Patients	164	100%
Median age (years, range)	32 (0.8-66)	
Diagnosis		
ALL	58	35%
AML	106	65%
Mortality	71	43%
Relapse	67	41%
GVHD		
acute GVHD I-II	46	28%
acute GVHD III-IV	9	5%
chronic GVHD	85	52%

ALL, acute lymphoblastic leukemia; AML, acute myeloid leukemia.

### Blood collection and genomic DNA extraction

The specimens for analysis were the remnant DNA for short tandem repeats (STR) analysis obtained from the Department of Laboratory Medicine. Briefly, peripheral blood (3 ml) was collected from patients in the blood collection tube with EDTA as the anticoagulant. The buffy coat was collected for genomic DNA extraction by using QIAamp DNA Blood Mini Kit according to the instruction of the manufacturer (Qiagen, Valencia, California, USA). The OD_260/280_ of the template genomic DNA had to be in the range of 1.8-2.0 with a minimal concentration of 100 ng/μl for genotyping and haplotyping by PCR.

### Selection of genotypes and haplotypes

Based on previous studies for the association of HLA-G genetic polymorphisms with the clinical outcomes of various diseases and organ transplantation (17–21, 23), the recipient genotypes and haplotypes in the 3’-UTR and 5’-URR of HLA-G gene were selected for analysis. For 3’-UTR, a total of 9 genetic variants including 8 SNPs and the 14-bp INDEL polymorphism (rs371194629) (**Table 2**), and 6 haplotypes (**Table 3**) which were classified according to the sequences of the 9 genetic variants (24) were selected for analysis. For 5’-URR, a total of 27 SNPs (**Table 2**) and 6 haplotypes (**Table 4**) which were classified according to the DNA sequences of the 27 SNPs were selected for analysis.

**Table 2:** The genetic variants of HLA-G gene for analysis in this study.

Genomic region	Genetic variants under analysis				
3’-UTR	rs371194629	rs1707	rs1710	rs17179101	rs17179108
	rs1063320	rs9380142	rs1610696	rs1233331	
5’-URR	rs1736936	rs1736935	rs3823321	rs1736934	rs17875389
	rs3115630	rs1632947	rs1632946	rs1233334	rs2249863
	rs2735022	rs35674592	rs17875391	rs1632944	rs201221694
	rs112940953	rs17875393	rs1736933	rs149890776	rs1736932
	rs17875394	rs17875395	rs17875396	rs1632943	rs191630481
	rs1233333	rs17875397			

**Table 3:** The haplotypes in the 3’-UTR of HLA-G under analysis in this study.

Haplotypes	Position, rs number, and sequences of the genetic variants									Frequency (n = 164)
	+2960	+2989	+2996	+3013	+3021	+3128	+3173	+3182	+3213	
	rs371194629	rs1707	rs1710	rs17179101	rs17179108	rs1063320	rs9380142	rs1610696	rs1233331	
UTR-1	Del	T	G	C	C	C	G	C	G	0.303
UTR-2	Ins	T	C	C	C	G	A	G	G	0.082
UTR-3	Del	T	C	C	C	G	A	C	G	0.252
UTR-4	Del	C	G	C	C	C	A	C	G	0.006
UTR-5	Ins	T	C	C	T	G	A	C	G	0.006
UTR-7	Ins	T	C	A	T	G	A	C	G	0.197

The A nucleotide of the ATG start codon was designated as position +1.

**Table 4:** The haplotypes in the 5’-URR of HLA-G under analysis in this study.

Haplotypes	Position, rs number and sequences of the genetic variants																											Frequency (n = 161)
	-1305	-1179	-1155	-1140	-1138	-1121	-964	-762	-725	-716	-689	-666	-646	-633	-545	-539	-509	-486	-483	-477	-443	-400	-391	-369	-355	-201	-56	
	rs1736936	rs1736935	rs3823321	rs1736934	rs17875389	rs3115630	rs1632947	rs1632946	rs1233334	rs2249863	rs2735022	rs35674592	rs17875391	rs1632944	rs201221694	rs112940953	rs17875393	rs1736933	rs149890776	rs1736932	rs17875394	rs17875395	rs17875396	rs1632943	rs191630481	rs1233333	rs17875397	
G010101a	G	A	G	A	A	C	G	C	C	T	A	G	A	G	5g	A	C	A	A	C	G	G	G	C	G	G	C	0.304
G010101b	G	A	G	A	A	C	G	C	G	T	A	G	A	G	5g	A	C	A	A	C	G	G	G	C	G	G	C	0.006
G010102a	A	G	G	T	A	C	A	T	C	G	G	T	A	A	5g	A	C	C	A	G	G	G	G	A	G	A	C	0.252
G0103a	G	G	G	A	G	C	G	C	T	T	A	G	A	G	6g	A	G	A	A	G	G	A	A	A	G	G	T	0.003
G0104a	A	G	A	A	A	C	A	T	C	G	G	T	A	A	5g	A	C	C	A	G	G	G	G	A	G	A	C	0.211
G0104b	A	G	A	A	A	C	A	T	C	G	G	T	A	A	5g	A	C	C	A	G	A	G	G	A	G	A	C	0.096

The first nucleotide at the 5’ of the ATG start codon was designated as position -1.

### PCR and sequencing

Primers (**Table 5**) were designed to amplify a 366-bp DNA fragment covering the 3’-UTR of HLA-G gene that contained the sequences of the selected 9 genetic variants and the 6 haplotypes. Primes (**Table 5**) were also designed to amplify a 1402-bp DNA fragment covering the 5’-URR of HLA-G gene that contained the sequences of the selected 27 SNPs and 6 haplotypes. The 1500 bp upstream of the start codon (ATG) was considered as the promoter region in the 5’-URR as described in most studies (25). PCR amplification was performed in a reaction mixture (25 μl) containing 0.2 mM of dNTP, 1.5 mM of MgCl_2_, 5 pmol of forward and reversed primers, 1 unit of Platinum Taq DNA polymerase (Invitrogen, Carlsbad, CA, USA), and 200 ng of genomic DNA. PCR was initiated at 94°C for 3 min, followed by 30 cycles of 94°C for 30 sec, 58°C for 30 sec, and 72°C for 90 sec, with a final extension step at 72°C for 10 min. The PCR products were verified by fractionating on a 2% agarose gel. The PCR amplicons were sequenced by using the Big Dye Terminator Cycle Sequencing kit (Thermo Fisher, Waltham, Massachusetts, USA) followed by analysis using the ABI PRISM genetic analyzer (Thermo Fisher, Waltham, Massachusetts, USA). Because of either insufficient genomic DNA or PCR failure, not every donor had complete data available.

**Table 5:** The primer pairs for amplifying the 3’-UTR and 5’-URR of HLA-G.

Genomic region	Primer sequences	PCR product (bp)
3’-UTR	5’-TCACCCCTCACTGTGACTGATA-3’	366
	5’-CTGTGGAAAGTTCTCATGTCTTCC-3’	
5’-URR	5’-GCCTGACATTCTAGAAGCTTCACAAGAAT-3’	1402
	5’-ATCCTTGGCGTCTGGGGAGAAT-3’	

### Definition of the outcomes post-HSCT

The definition of outcomes post-HSCT has been described previously (26). Briefly, mortality was referred to the state of patients who died in the duration of study. Relapse was defined as recurrence of malignancy based on the overall evaluation of one or more of the following test results: bone marrow morphology, minimal residual disease by either flow cytometry, cytogenetics, imaging results, or STR analysis with the presence of > 5% recipient STR alleles in the chimeric test (27). GVHD was considered as acute GVHD (aGVHD) and chronic GVHD (cGVHD) when it occurs within 100 days after transplantation and more than 100 days after transplantation or continually for more than 100 days without remission, respectively (5). aGVHD was divided into four grades (Grade I, II, III, and IV) according to the clinical characteristics of organs (26) Grades I–II were defined as mild GVHD, Grades III–IV were defined as severe GVHD, and patients without aGVHD or cGVHD during the study period were defined as no GVHD. Patients with any forms of GVHD was defined when patients were present with either aGVHD or cGVHD.

### Statistical analysis

Patients were grouped as case or control for statistical analyses of the association between the genotype and haplotype of patients and the effectiveness of HSCT. For mortality analysis, patients who died and lived at the end of this study were grouped as case and control, respectively. For relapse analysis, patients who relapsed and did not relapse at the end of this study were grouped as the case and control, respectively. For GVHD analysis, patients who developed stages I-II aGVHD, stages III-IV of aGVHD, cGVHD, and any forms of GVHD were considered as case and patients without development of GVHD were considered as control.

The PLINK software was used for genotyping analysis (http://pngu.mgh.harvard.edu/~purcell/plink/index.shtml). The associations of genotypes and haplotypes in the 3’-UTR and 5’-URR of HLA-G gene with the outcomes of patients post-HSCT (i.e. Grade I-II aGVHD, Grade III-IV aGVHD, cGVHD, any forms of GVHD, mortality, and relapse) were evaluated by analysis of genotype groups (AA, Aa, and aa) using various genetic models including additive, homozygous, heterozygous, dominant and recessive models and logistic regression analysis as described previously (26). Briefly, the allele with higher frequency was referred to major allele “A” and the other was minor allele “a”. For analysis using the additive model, “AA”, “Aa”, and “aa” were compared to each other (AA vs. Aa vs. aa). For homozygous model, the “AA” was compared to “aa”. For the heterozygous model, the “AA” was compared to “Aa”. For the dominant model, the collective genotypes (“Aa” + “aa”) were compared to a reference genotype “AA”. For the recessive model, “aa” is compared to a collective (“AA” + “Aa”) reference genotype.

The haplotypes with frequency less than 1% were excluded from the analysis. The dosage of a specific haplotype was first defined as 2, 1, and 0 when the indicated haplotype was homozygous, heterozygous, and not present in the recipient genomic DNA, respectively. Analysis was performed by either heterozygous or homozygous haplotype models. For heterozygous haplotype model, the occurrence of adverse outcomes for patients with two copies of the same haplotypes were compared with patients with one copy of the haplotype. For homozygous haplotype model, the occurrence of adverse outcomes for patients with two copies of the same haplotypes were compared with patients without the specific haplotype.

The SPSS 17.0 software was used for statistical analysis. A p < 0.05 was considered as statistical significance for all tests. Odds ratio (OR) and 95% confidence interval (CI) were also provided.

## Results

### Association of genetic polymorphisms in the 3’-UTR and 5’-URR of HLA-G gene with the risk of adverse outcomes for patients with leukemia post-HSCT

A total of 9 genetic polymorphisms in the 3’-UTR of HLA-G gene were analyzed to determine the association of these genotypes with the risks of adverse outcomes post-HSCT, including mortality, disease relapse, and GVHD. The complete genotype and allele frequency datasets for all recipients are summarized in the **Supplementary Tables S1**-**S4**. The genetic polymorphisms that were significantly associated with the risk of adverse outcomes are shown in **Tables 6**–**9**. Our data revealed that genetic polymorphisms in the 3’-UTR of HLA-G gene were only associated with the risks for development of GVHD, but not the mortality and disease relapse post- HSCT (**Table 6**; **Supplementary Table S1**). By analysis using the heterozygous model, patients with del/ins genotype of the 14-bp INDEL (rs371194629) were associated with an increased risk of cGVHD (OR = 2.98, 95% CI = 1.10 – 8.07, p = 0.03) when compared to patients with del/del genotype. By analysis using the dominant model, patients with the del/ins and ins/ins genotypes of the 14-bp INDEL were associated with an increased risk of cGVHD (OR = 3.15, 95% CI = 1.21 – 8.18, p = 0.02) or any form of GVHD (OR = 2.52, 95% CI = 1.01 – 6.26, p = 0.04) when compared to patients with del/del genotype. As revealed by analysis using the heterozygous model, patients with A/G genotype of rs9380142 had a higher risk to develop cGVHD (OR = 3.01, 95% CI = 1.08-8.42, p = 0.03) or any form of GVHD (OR = 2.62, 95% CI = 0.98 – 7.03, p = 0.05) when compared to patients with A/A genotype.

**Table 6:** Association of genetic variants in 3’-UTR of HLA-G with the adverse outcomes post-HSCT.

SNP	No. of patients (%)			Additive p	Model	Logistic regression p	OR (95% CI)
Chronic GVHD							
rs371194629	del/del	del/ins	ins/ins	0.05	Heterozygous	0.03*	2.98 (1.10-8.07)
Cases^a^	33	43	9		Homozygous	0.25	4.36 (0.51-37.48)
Controls^a^	16	7	1		Dominant	0.02*	3.15 (1.21-8.18)
					Recessive	0.45	2.72 (0.33-22.65)
rs9380142	A/A	A/G	G/G	0.08	Heterozygous	0.03*	3.01 (1.08-8.42)
Cases	29	47		9	Homozygous	1.00	1.01 (0.26-3.88)
Controls	13	7	4		Dominant	0.08	2.28 (0.91-5.73)
					Recessive	0.48	0.59 (0.17-2.12)
Any form of GVHD							
rs371194629	del/del	del/ins	ins/ins	0.12	Heterozygous	0.07	2.40 (0.92-6.22)
Cases	62	65	13		Homozygous	0.45	3.36 (0.41-27.59)
Controls	16	7	1		Dominant	0.04*	2.52 (1.01-6.26)
					Recessive	0.70	2.35 (0.29-18.88)
rs9380142	A/A	A/G	G/G	0.13	Heterozygous	0.05*	2.62 (0.98-7.03)
Cases	51	72	17		Homozygous	1.00	1.08 (0.31-3.77)
Controls	13	7	4		Dominant	0.10	2.06 (0.86-4.94)
					Recessive	0.52	0.69 (0.21-2.27)

a Cases: patients with the event; Controls: patients without GVHD.*, p < 0.05.

The association of 27 genetic polymorphisms in 5’-URR of HLA-G gene with post-HSCT adverse outcomes of patients were also investigated. Because of the insufficient amount of genomic DNA for PCR, the samples of three patients including one patient with ALL (the patient had mortality and chronic GVHD, but without relapse) and two patients with AML (both patients had mortality and chronic GHVD, and one had relapse) failed to be tested at 5’-URR. No association of these polymorphisms with mortality and relapse was found (**Table 7**). As revealed by analysis using the additive model, the genotype frequency (G/G vs. A/G vs. A/A) of rs3823321 was significantly different between cGVHD and no GVHD (p < 0.01) as well as between any forms of GVHD and no GVHD (p < 0.01). By analysis using the homozygous model, the A/A genotype of rs3823321 had 0.15 (95% CI = 0.04 - 0.53, p < 0.01) and 0.23 times (95% CI = 0.08 - 0.69, p = 0.01) lower risk for development of cGVHD and any forms of GVHD, respectively, when compared to the G/G genotype. By analysis using recessive model, the A/A genotype of rs3823321 was also associated with a lower risk for development of cGVHD (OR = 0.16, 95% CI = 0.05 – 0.52, p < 0.01) and any forms of GVHD (OR = 0.23, 95% CI = 0.08 – 0.63, p = 0.01). Analysis by heterozygous model revealed that patients with A/A genotype of rs1736934 had an increased risk of cGVHD when compared to patients with T/A genotype (A/A vs. A/T; OR = 2.77, 95% CI = 1.02 - 7.54, p = 0.04). As revealed by analysis with dominant model, patients with T/T and A/T genotypes of rs1736934 had 2.97 times higher risk for development of cGVHD (A/A vs. A/T + T/T; 95% CI =1.14-7.73, p = 0.02) when compared to patients with A/A genotype.

**Table 7:** Association of genetic variants in 5’-URR of HLA-G with the adverse outcomes post-HSCT.

SNP	No. of patients (%)				Additive p	Model	Logistic regression p	OR (95% CI)
Chronic GVHD								
rs3823321	G/G		A/G	A/A	<0.01*	Heterozygous	0.75	0.84 (0.28-2.49)
Cases	46		30	6		Homozygous	<0.01*	0.15 (0.04-0.53)
Controls	9		7	8		Dominant	0.11	0.47 (0.18-1.20)
						Recessive	<0.01*	0.16 (0.05-0.52)
rs1736934	A/A		A/T	T/T	0.07	Heterozygous	0.04*	2.77 (1.02-7.54)
Cases	33		40	9		Homozygous	0.25	4.36 (0.51-37.48)
Controls	16		7	1		Dominant	0.02*	2.97 (1.14-7.73)
						Recessive	0.45	2.84 (0.34-23.59)
Any form of GVHD								
rs3823321	G/G		A/G	A/A	0.01*	Heterozygous	0.99	1.01 (0.35-2.88)
Cases	69		54	14		Homozygous	0.01*	0.23 (0.08-0.69)
Controls	9		7	8		Dominant	0.25	0.59 (0.24-1.44)
						Recessive	0.01*	0.23 (0.08-0.63)

Cases: patients with the event; Controls: patients without GVHD.*, p < 0.05.

### Association of 3’-UTR and 5’-URR haplotypes of HLA-G gene with the risk for post-HSCT adverse outcomes of patients with leukemia

The association of haplotypes in 3’-UTR with the adverse outcomes of patients receiving HSCT was further analyzed. Six different haplotypes including UTR-1, UTR-2, UTR-3, UTR-4, UTR-5, and UTR-7 were classified according to the sequence combination of 8 SNPs (rs371194629, rs1707, rs1710, rs17179101, rs17179108, rs1063320, rs9380142, rs1610696, and rs1233331) located in 3’-UTR. Among patients under analysis, the frequency of UTR-1 (30.3%) was the highest followed by UTR-3 (25.2%) and UTR-7 (19.7%) (**Table 3**).

During haplotype analysis (**Table 8**; **Supplementary Table S3**), the dosage of a haplotype was defined as 2, 1, and 0 when the indicated haplotype was homozygous, heterozygous, and not present in the recipient genomic DNA, respectively. As revealed by additive analysis, the dosage of UTR-3 haplotype was reversely associated with the development of cGVHD (p < 0.01) and any forms of GVHD (p = 0.01). Patients with heterozygous UTR-3 and without UTR-3 had 5.71 times (95% CI = 1.50 - 21.84, p = 0.01), and 7.26 times higher risk (95% CI = 2.03 - 25.98, p < 0.01) for development of cGVHD when compared to patients with homozygous UTR-3, respectively. In addition, patients with heterozygous UTR-3 and without UTR-3 had 4.84 times (95% CI = 1.49 - 15.75, p = 0.02) and 4.92 times (95% CI = 1.61 - 15.10, p = 0.01) higher risk for development of any forms of GVHD when compared to patients with homozygous UTR-3, respectively.

**Table 8:** Association of haplotypes in 3’-UTR of HLA-G with adverse outcomes post-HSCT.

Haplotype	No. of patients			Additive P value	Model	Logistic regression P value	OR (95% CI)
	2 ^a^	1 ^a^	0 ^a^				
Chronic GVHD							
UTR-3							
Cases	6	30	49	<0.01*	Heterozygous (2 vs 1)	0.01*	5.71 (1.50-21.84)
Controls	8	7	9		Homozygous (2 vs 0)	<0.01*	7.26 (2.03-25.98)
Any form of GVHD							
UTR-3							
Cases	13	55	72	0.01*	Heterozygous (2 vs 1)	0.02*	4.84 (1.49-15.75)
Controls	8	7	9		Homozygous (2 vs 0)	0.01*	4.92 (1.61-15.10)

a Dose of haplotype: 2 means that the haplotype of UTR-3 is present in both strands of DNA. 1 means that the haplotype of UTR-3 is present in one strand of DNA, and the other strand of DNA carries the other type of haplotype. 0 means that the haplotype of UTR-3 is absence in both strands of DNA.

Cases: patients with the event; Controls: patients without GVHD.*, p < 0.05.

The association of haplotypes in 5’-URR with the adverse outcomes of patients receiving HSCT was also analyzed (**Table 9**; **Supplementary Table S4**). Patients enrolled in this study had 6 different haplotypes including 010101a, 010101b, 010102a, 0103a, 0104a, and 0104b. These haplotypes were classified according to the sequence combination of the 27 SNPs located in the 5’-URR including rs1736936, rs1736935, rs3823321, rs1736934, rs17875389, rs3115630, rs1632947, rs1632946, rs1233334, rs2249863, rs2735022, rs35674592, rs17875391, rs1632944, rs201221694, rs112940953, rs17875393, rs1736933, rs149890776, rs1736932, rs17875394, rs17875395, rs17875396, rs1632943, rs191630481, rs1233333, and rs17875397. Among the patients under analysis, the frequency of 010101a (30.4%) was the highest followed by 010102a (25.2%) and 0104a (21.1%).

**Table 9:** Association of haplotypes in 5’-URR of HLA-G with adverse outcomes post-HSCT.

Haplotype	No. of patients			Additive (P value)	Model	Logistic regression (P value)	OR (95% CI)
	2 ^a^	1 ^a^	0 ^a^				
Chronic GVHD							
0104a							
Cases	2	22	58	<0.01*	Heterozygous (2 vs 1)	0.01*	9.43 (1.54-57.74)
Controls	6	7	11		Homozygous (2 vs 0)	<0.01*	15.82 (2.82-88.80)
Any form of GVHD							
0104a							
Cases	6	39	92	<0.01*	Heterozygous (2 vs 1)	0.02*	5.57 (1.39-22.33)
Controls	6	7	11		Homozygous (2 vs 0)	<0.01*	8.36 (2.30-30.47)

a Dose of haplotype: 2 means that the haplotype of 0140a is present in both strands of DNA. 1 means that the haplotype of 0104a is present in one strand of DNA, and the other strand of DNA carries the other type of haplotype. 0 means that the haplotype of 0104a is absence in both strands of DNA.

Cases: patients with the event; Controls: patients without GVHD.*, p < 0.05.

As revealed by additive analysis, the dosage of 0104a haplotype was reversely associated with the development of cGVHD (p < 0.01) and any forms of GVHD (p < 0.01) post-HSCT. Patients with heterozygous 0104a and without 0104a had 9.43 times (95% CI = 1.54 - 57.74, p = 0.01) and 15.82 times (95% CI = 2.82 - 88.80, p < 0.01) higher risk for development of cGVHD when compared to patients with homozygous 0104a, respectively. Patients with heterozygous 0104a and without 0140a had 5.57 times (95% CI = 1.39 - 22.33, p = 0.02) and 8.36 times (95% CI = 2.30 - 30.47, p < 0.01) higher risk for development of any forms of GVHD when compared to patients with homozygous 0104a, respectively.

## Discussion

Genetic polymorphisms and haplotypes of HLA-G have been reported to associate with various diseases (9). The genetic polymorphisms and haplotypes in the 3’-UTR and 5’-URR of HLA-G gene were selected for analysis because genetic variants in these regions have been shown to associate with clinical outcomes of various diseases and organ transplantation (17–21, 23). In this study, we investigated whether the SNPs and haplotypes in the 3’-UTR and 5’-URR of HLA-G are associated with the occurrence of adverse outcomes (mortality, relapse, and GVHD) for patients with AML and ALL. Our data revealed that 2 SNPs and 1 haplotype located in the 3’-UTR and 2 SNPs and 1 haplotype located in the 5’-URR of HLA-G were associated with the occurrence of cGVHD or any forms of GVHD. No SNPs was associated with mortality and relapse. This study provides new insights on the selection of donor/recipient pair when performing HSCT for therapy of patients with AML and ALL.

There are soluble and membrane-bound forms of HLA-G. Through alternative splicing, three major soluble forms of HLA-G (HLA-G5, -G6, and -G7) are expressed in the body fluid such as saliva, plasma, and cerebrospinal fluid (7). HLA-G is also expressed as the membrane-bound form in the placenta trophoblast cells, thymus, peripheral blood mononuclear cells, cornea, and mesenchymal stem cells (7). Soluble and membrane-bound HLA-G interacts with different types of receptors on immune cells and causes negative intracellular signaling leading to a decrease in the inflammatory response of the immune system. Previous study by Boukouaci et al. has demonstrated that patients with low expression of HLA-G have an increased risk of severe aGVHD after HSCT (13). The concentration of the soluble HLA-G in patients after HSCT was also related to the risk and grade of aGVHD (28, 29). Because genetic polymorphism in the untranslated or promoter region may regulate the levels of HLA-G gene expression and alter the status of immune response and the magnitude of immune tolerance, the SNPs and haplotypes locate in the 3’-UTR and 5’-URR were selected in this study to define whether these SNPs and haplotypes are associated with the mortality, the risk of relapse, and the development of GVHD for patients receiving HSCT.

According to the findings of this study, genetic polymorphisms of rs371194629 and rs9380142, and the UTR-3 haplotype located in the 3’-UTR were associated with the risk for the development of cGVHD and any forms of GVHD. These polymorphisms have been shown to involve in the regulation of HLA-G expression (30). Higher HLA-G expression usually causes suppression of immune response and leads to a better immune tolerance. Accordingly, the 14-bp INDEL fragment (5’-ATTTGTTCATGCCT-3’) of rs371194629 is associated with the splicing and the stability of the mRNA, because it contains an AUUUG domain that putatively elicits an AU-pentamer-like effect leading to a decrease in mRNA stability. Hence, the DEL allele provides a higher stability of the mRNA, associated with a high expression of HLA-G (31). The A/G allele of rs9380142 is also implicated in the stability of HLA-G mRNA. The A allele at this position modifies an AU-rich motif and decreases the stability of the corresponding mRNA, while the G allele at this position is associated with an increase in the production of HLA-G (32). Moreover, the UTR-3 haplotype had a 14-bp deletion in rs371194629, an A allele in rs9380142, and a G allele in rs1063320. The presence of a G allele in rs1063320 of the UTR-3 haplotype increases the binding of miR-148a, miR148b and miR-152 to this region and causes degradation of the primary transcript and suppression of its translation, thereby decreasing the production of HLA-G protein. In contrast, the presence of C allele at this position causes a decrease in affinity to the aforementioned miRNA, and an increase in the mRNA availability and the production of HLA-G (15).

Similar to our findings, the association of rs371194629 with the development of GVHD and other post-transplant complications have been reported (33, 34). La Nasa et al. found that in a cohort of 53 thalassemia patients transplanted from an unrelated donor, patients who are homozygous for the 14-bp deletion have a higher risk to develop aGVHD than patients who are homozygous for the 14-bp insertion (34). On the other hand, patients with A/G genotype of rs9380142 had an increased risk for occurrence of cGVHD and any forms of GVHD. Martelli-Palomino et al. demonstrated that individuals with A/G genotype of rs9380142 had higher soluble levels of HLA-G protein when compared to individuals with other genotypes, although significance was not reached (22). In haplotype analysis, we found that UTR-3 was a protective factor for GVHD development. UTR-3 had a 14-bp deletion in rs371194629 and an A allele in rs9380142. The haplotypes of 3’-UTR were associated with the level of soluble HLA-G (22). Poras et al. confirmed that 3’-UTR haplotypes alter the expression of HLA-G by luciferase reporter assay, although the effect is moderate (35). The study by Shivakumar et al. showed that the allele with 14-bp deletion in rs371194629 was associated with reduced IL-6 gene expression and the risk of schizophrenia (36). We have noted that these findings are not always consistent with the hypothesis that patients with higher HLA-G expression tend to have a better immune tolerance leading to a lower risk for the occurrence of cGVHD or any forms of GVHD. The reasons for the discrepancy are not clear. Because the incidence for the polymorphisms is different among different ethnical population and gender (37), these factors may be considered as variates in future analysis. Alternatively, the complexity of HLA system and different analytical methods among different studies may contribute to the inconsistency among the studies. It is also likely that the polymorphism in HLA-G 3’-UTR may associate with GVHD through regulating other factors, such as altering IL-6 gene expression, rather than directly altering the expression of HLA-G (36). Further study is required to investigate whether affecting the levels of HLA-G expression is the major effects of HLA-G polymorphisms on immune tolerance and the occurrence of GVHD.

We further demonstrated in this study that the genotypes of rs3823321 and rs1736934 and the G0104a haplotype located at the 5’-URR of HLA-G gene are associated with the risk for occurrence of cGVHD and development of any forms of GVHD. Patients with G/G genotype of rs3823321 is associated with higher risk for cGVHD as well as development of any forms of GVHD, while T-allele of rs1736934 is associated with higher risk for cGVHD. Notably, G0104a haplotype had the A-allele at rs3823321 and rs1736934. The roles of 5’-URR in the regulation of HLA-G gene expression and in immune tolerance and disease development are not yet well studies. Previous study showed that rs3823321 G was a binding site for SOX5 and progesterone receptor, and rs1736934 T is a binding site for POU2F1 (38). These transcription factors have been shown to play a role in regulating HLA-G expression (38). Progesterone has also been shown to enhance HLA-G expression (39, 40). With the A allele in G0104a haplotype, the binding of these transcription factors to HLA-G promoter may be impaired in patients with G0104a haplotype leading to lower HLA-G expression when compared to non-G0104a haplotypes. This may explain why patients with G0104a are associated with lower risk for development of any forms of GVHD and cGVHD. Nevertheless, the underlying mechanisms remain to be elucidated and require further investigation.

In conclusion, this study showed that six genotypes and haplotypes in 3’-UTR and 5’-URR of HLA-G gene were associated with the risk for development of any forms of GVHD and cGVHD in patients post-HSCT. The findings of this study are applicable to predict the risk of adverse outcomes post-HSCT that allow physicians to prescribe preventive plan or treatment for patients on time. Moreover, effective prevention of GVHD is depended on the induction of peripheral immune tolerance. The role of HLA-G gene polymorphism in the regulation of immune tolerance and the development of GVHD post-HSCT is worthy to investigate further.

## Data availability statement

The datasets presented in this study can be found in online repositories. The names of the repository/repositories and accession number(s) can be found in **Supplementary Table 5**.

## Ethics statement

The studies involving human participants were reviewed and approved by Institutional Review Board (IRB) of Chang Gung Memorial Hospital. The patients/participants provided their written informed consent to participate in this study.

## Author contributions

D-PC conceived and designed the experiments and reviewed the final draft. P-NW contributed the materials. A-LH, D-PC, and C-PT analyzed and interpreted data. D-PC, F-PH, W-TL, and C-PT wrote draft of the manuscript. W-TW performed the experiments. All authors contributed to the article and approved the submitted version.
