# Supplementary material for: The association between genetic variants at 3’-UTR and 5’-URR of HLA-G gene and the clinical outcomes of patients with leukemia receiving hematopoietic stem cell transplantation
Source: Front Immunol. 2023 Feb 23;14:1093514. doi: 10.3389/fimmu.2023.1093514 (PMC9995383; doi:10.3389/fimmu.2023.1093514)
Supplement: Supplementary file 1 [file Table_1.doc]

**Supplementary Table S1.** Association study of the genetic variants in 3’-UTR of HLA-G with various adverse outcomes post-HSCT.

| **SNP** | **No. of patients (%)** | | | **Additive p** | **Model** | **Logistic regression p** | **OR (95 % CI)** |
| --- | --- | --- | --- | --- | --- | --- | --- |
| Mortality |  |  |  |  |  |  |  |
| rs371194629 | del/del | del/ins | ins/ins | 0.97 | Heterozygous | 0.79 | 1.09 (0.57-2.08) |
| Cases | 33 | 32 | 6 |  | Homozygous | 0.97 | 1.02 (0.32-3.23) |
| Controls | 45 | 40 | 8 |  | Dominant | 0.81 | 1.08 (0.58-2.00) |
|  |  |  |  |  | Recessive | 0.97 | 0.99 (0.32-2.97) |
|  |  |  |  |  |  |  |  |
| rs1707 | T/T | C/T | C/C | 1.00 | Heterozygous | 1.00 | 1.31 (0.08-21.38) |
| Cases | 70 | 1 | 0 |  | Homozygous | NA | NA |
| Controls | 92 | 1 | 0 |  | Dominant | 1.00 | 1.31 (0.08-21.38) |
|  |  |  |  |  | Recessive | NA | NA |
|  |  |  |  |  |  |  |  |
| rs1710 | C/C | C/G | G/G | 0.98 | Heterozygous | 0.98 | 1.01 (0.52-1.97) |
| Cases | 27 | 34 | 10 |  | Homozygous | 0.83 | 1.11 (0.42-2.95) |
| Controls | 36 | 45 | 12 |  | Dominant | 0.93 | 1.03 (0.55-1.94) |
|  |  |  |  |  | Recessive | 0.83 | 1.11 (0.45-2.73) |
|  |  |  |  |  |  |  |  |
| rs17179101 | C/C | A/C | A/A | 0.59 | Heterozygous | 0.70 | 0.55 (0.10-2.99) |
| Cases | 42 | 27 | 2 |  | Homozygous | 0.52 | 1.24 (0.65-2.40) |
| Controls | 58 | 30 | 5 |  | Dominant | 0.68 | 1.14 (0.61-2.15) |
|  |  |  |  |  | Recessive | 0.70 | 0.51 (0.10-2.71) |
|  |  |  |  |  |  |  |  |
| rs17179108 | C/C | C/T | T/T | 0.68 | Heterozygous | 0.72 | 1.13 (0.59-2.16) |
| Cases | 42 | 27 | 2 |  | Homozygous | 0.70 | 0.54 (0.10-2.88) |
| Controls | 56 | 32 | 5 |  | Dominant | 0.89 | 1.05 (0.56-1.96) |
|  |  |  |  |  | Recessive | 0.70 | 0.51 (0.10-2.71) |
|  |  |  |  |  |  |  |  |
| rs1063320 | G/G | C/G | C/C | 0.98 | Heterozygous | 0.98 | 1.01 (0.52-1.97) |
| Cases | 27 | 34 | 10 |  | Homozygous | 0.83 | 1.11 (0.42-2.95) |
| Controls | 36 | 45 | 12 |  | Dominant | 0.93 | 1.03 (0.55-1.94) |
|  |  |  |  |  | Recessive | 0.83 | 1.11 (0.45-2.73) |
|  |  |  |  |  |  |  |  |
| rs9380142 | A/A | A/G | G/G | 0.97 | Heterozygous | 0.80 | 1.09 (0.56-2.12) |
| Cases | 27 | 35 | 9 |  | Homozygous | 0.96 | 1.03 (0.38-2.78) |
| Controls | 37 | 44 | 12 |  | Dominant | 0.82 | 1.08 (0.57-2.03) |
|  |  |  |  |  | Recessive | 0.97 | 0.98 (0.39-2.47) |
|  |  |  |  |  |  |  |  |
| rs1610696 | C/C | C/G | G/G | 0.58 | Heterozygous | 0.58 | 1.27 (0.55-2.89) |
| Cases | 58 | 13 | 0 |  | Homozygous | NA | NA |
| Controls | 79 | 14 | 0 |  | Dominant | 0.58 | 1.27 (0.55-2.89) |
|  |  |  |  |  | Recessive | NA | NA |
|  |  |  |  |  |  |  |  |
| rs1233331 | G/G | A/G | A/A | NA | Heterozygous | NA | NA |
| Cases | 71 | 0 | 0 |  | Homozygous | NA | NA |
| Controls | 93 | 0 | 0 |  | Dominant | NA | NA |
|  |  |  |  |  | Recessive | NA | NA |
|  |  |  |  |  |  |  |  |
| Relapse |  |  |  |  |  |  |  |
| rs371194629 | del/del | del/ins | ins/ins | 0.23 | Heterozygous | 0.45 | 0.78 (0.41-1.49) |
| Cases | 31 | 33 | 3 |  | Homozygous | 0.19 | 2.42 (0.62-9.37) |
| Controls | 47 | 39 | 11 |  | Dominant | 0.78 | 0.92 (0.49-1.71) |
|  |  |  |  |  | Recessive | 0.12 | 2.73 (0.73-10.18) |
|  |  |  |  |  |  |  |  |
| rs1707 | T/T | C/T | C/C | 0.51 | Heterozygous | 0.51 | NA |
| Cases | 67 | 0 | 0 |  | Homozygous | NA | NA |
| Controls | 95 | 2 | 0 |  | Dominant | 0.51 | NA |
|  |  |  |  |  | Recessive | NA | NA |
|  |  |  |  |  |  |  |  |
| rs1710 | C/C | C/G | G/G | 0.14 | Heterozygous | 0.17 | 0.62 (0.32-1.22) |
| Cases | 23 | 38 | 6 |  | Homozygous | 0.43 | 1.53 (0.53-4.47) |
| Controls | 40 | 41 | 16 |  | Dominant | 0.37 | 0.75 (0.39-1.42) |
|  |  |  |  |  | Recessive | 0.16 | 2.01 (0.74-5.43) |
|  |  |  |  |  |  |  |  |
| rs17179101 | C/C | A/C | A/A | 0.20 | Heterozygous | 0.25 | 3.84 (0.45-33.09) |
| Cases | 39 | 27 | 1 |  | Homozygous | 0.31 | 0.71 (0.37-1.37) |
| Controls | 61 | 30 | 6 |  | Dominant | 0.55 | 0.82 (0.44-1.55) |
|  |  |  |  |  | Recessive | 0.24 | 4.35 (0.51-37.01) |
|  |  |  |  |  |  |  |  |
| rs17179108 | C/C | C/T | T/T | 0.19 | Heterozygous | 0.29 | 0.70 (0.37-1.35) |
| Cases | 38 | 28 | 1 |  | Homozygous | 0.25 | 3.80 (0.44-32.81) |
| Controls | 60 | 31 | 6 |  | Dominant | 0.51 | 0.81 (0.43-1.52) |
|  |  |  |  |  | Recessive | 0.24 | 4.35 (0.51-37.01) |
|  |  |  |  |  |  |  |  |
| rs1063320 | G/G | C/G | C/C | 0.14 | Heterozygous | 0.17 | 0.62 (0.32-1.22) |
| Cases | 23 | 38 | 6 |  | Homozygous | 0.43 | 1.53 (0.53-4.47) |
| Controls | 40 | 41 | 16 |  | Dominant | 0.37 | 0.75 (0.39-1.42) |
|  |  |  |  |  | Recessive | 0.16 | 2.01 (0.74-5.43) |
|  |  |  |  |  |  |  |  |
| rs9380142 | A/A | A/G | G/G | 0.16 | Heterozygous | 0.14 | 0.61 (0.31-1.19) |
| Cases | 23 | 38 | 6 |  | Homozygous | 0.54 | 1.40 (0.48-4.11) |
| Controls | 41 | 41 | 15 |  | Dominant | 0.31 | 0.71 (0.37-1.36) |
|  |  |  |  |  | Recessive | 0.22 | 1.86 (0.68-5.07) |
|  |  |  |  |  |  |  |  |
| rs1610696 | C/C | C/G | G/G | 0.38 | Heterozygous | 0.38 | 1.17 (0.62-3.50) |
| Cases | 58 | 9 | 0 |  | Homozygous | NA | NA |
| Controls | 79 | 18 | 0 |  | Dominant | 0.38 | 1.17 (0.62-3.50) |
|  |  |  |  |  | Recessive | NA | NA |
|  |  |  |  |  |  |  |  |
| rs1233331 | G/G | A/G | A/A | NA | Heterozygous | NA | NA |
| Cases | 67 | 0 | 0 |  | Homozygous | NA | NA |
| Controls | 97 | 0 | 0 |  | Dominant | NA | NA |
|  |  |  |  |  | Recessive | NA | NA |
|  |  |  |  |  |  |  |  |
| Grades I–II GVHD |  |  |  |  |  |  |  |
| rs371194629 | del/del | del/ins | ins/ins | 0.61 | Heterozygous | 0.36 | 1.65 (0.56-4.82) |
| Cases | 25 | 18 | 3 |  | Homozygous | 1.00 | 1.92 (0.18-20.10) |
| Controls | 16 | 7 | 1 |  | Dominant | 0.32 | 1.68 (0.60-4.70) |
|  |  |  |  |  | Recessive | 1.00 | 1.61(0.16-16.31) |
|  |  |  |  |  |  |  |  |
| rs1707 | T/T | C/T | C/C | 0.34 | Heterozygous | 0.34 | NA |
| Cases | 46 | 0 | 0 |  | Homozygous | NA | NA |
| Controls | 23 | 1 | 0 |  | Dominant | 0.34 | NA |
|  |  |  |  |  | Recessive | NA | NA |
|  |  |  |  |  |  |  |  |
| rs1710 | C/C | C/G | G/G | 0.51 | Heterozygous | 0.27 | 1.83 (0.62-5.45) |
| Cases | 18 | 22 | 6 |  | Homozygous | 1.00 | 1.00 (0.23-4.31) |
| Controls | 12 | 8 | 4 |  | Dominant | 0.38 | 1.56 (0.58-4.21) |
|  |  |  |  |  | Recessive | 0.73 | 0.75 (0.19-2.96) |
|  |  |  |  |  |  |  |  |
| rs17179101 | C/C | A/C | A/A | 0.58 | Heterozygous | 1.00 | 1.24 (0.11-14.70) |
| Cases | 29 | 15 | 2 |  | Homozygous | 0.29 | 1.86 (0.58-6.00) |
| Controls | 18 | 5 | 1 |  | Dominant | 0.31 | 1.76 (0.59-5.29) |
|  |  |  |  |  | Recessive | 1.00 | 1.05 (0.09-12.15) |
|  |  |  |  |  |  |  |  |
| rs17179108 | C/C | C/T | T/T | 0.38 | Heterozygous | 0.16 | 2.27 (0.71-7.24) |
| Cases | 27 | 17 | 2 |  | Homozygous | 1.00 | 1.33 (0.11-15.82) |
| Controls | 18 | 5 | 1 |  | Dominant | 0.18 | 2.11 (0.71-6.31) |
|  |  |  |  |  | Recessive | 1.00 | 1.05 (0.09-12.15) |
|  |  |  |  |  |  |  |  |
| rs1063320 | G/G | C/G | C/C | 0.51 | Heterozygous | 0.27 | 1.83 (0.62-5.45) |
| Cases | 18 | 22 | 6 |  | Homozygous | 1.00 | 1.00 (0.23-4.31) |
| Controls | 12 | 8 | 4 |  | Dominant | 0.38 | 1.56 (0.58-4.21) |
|  |  |  |  |  | Recessive | 0.73 | 0.75 (0.19-2.96) |
|  |  |  |  |  |  |  |  |
| rs9380142 | A/A | A/G | G/G | 0.32 | Heterozygous | 0.14 | 2.27 (0.75-6.89) |
| Cases | 18 | 22 | 6 |  | Homozygous | 1.00 | 1.08 (0.25-4.63) |
| Controls | 13 | 7 | 4 |  | Dominant | 0.23 | 1.84 (0.68-4.99) |
|  |  |  |  |  | Recessive | 0.73 | 0.75 (0.19-2.96) |
|  |  |  |  |  |  |  |  |
| rs1610696 | C/C | C/G | G/G | 1.00 | Heterozygous | 1.00 | 0.77 (0.12-4.94) |
| Cases | 43 | 3 | 0 |  | Homozygous | NA | NA |
| Controls | 22 | 2 | 0 |  | Dominant | 1.00 | 0.77 (0.12-4.94) |
|  |  |  |  |  | Recessive | NA | NA |
|  |  |  |  |  |  |  |  |
| rs1233331 | G/G | A/G | A/A | NA | Heterozygous | NA | NA |
| Cases | 46 | 0 | 0 |  | Homozygous | NA | NA |
| Controls | 24 | 0 | 0 |  | Dominant | NA | NA |
|  |  |  |  |  | Recessive | NA | NA |
|  |  |  |  |  |  |  |  |
| Grades III–IV GVHD |  |  |  |  |  |  |  |
| rs371194629 | del/del | del/ins | ins/ins | 0.47 | Heterozygous | 0.41 | 2.29 (0.44-11.86) |
| Cases | 4 | 4 | 1 |  | Homozygous | 0.41 | 4.00 (0.20-78.79) |
| Controls | 16 | 7 | 1 |  | Dominant | 0.43 | 2.50 (0.52-11.96) |
|  |  |  |  |  | Recessive | 0.48 | 2.88 (0.16-51.53) |
|  |  |  |  |  |  |  |  |
| rs1707 | T/T | C/T | C/C | 1.00 | Heterozygous | 1.00 | NA |
| Cases | 9 | 0 | 0 |  | Homozygous | NA | NA |
| Controls | 23 | 1 | 0 |  | Dominant | 1.00 | NA |
|  |  |  |  |  | Recessive | NA | NA |
|  |  |  |  |  |  |  |  |
| rs1710 | C/C | C/G | G/G | 0.93 | Heterozygous | 1.00 | 1.13 (0.20-6.43) |
| Cases | 4 | 3 | 2 |  | Homozygous | 1.00 | 1.50 (0.20-11.54) |
| Controls | 12 | 8 | 4 |  | Dominant | 1.00 | 1.25 (0.27-5.83) |
|  |  |  |  |  | Recessive | 1.00 | 1.43 (0.21-9.58) |
|  |  |  |  |  |  |  |  |
| rs17179101 | C/C | A/C | A/A | 0.52 | Heterozygous | 0.43 | 3.60 (0.19-68.34) |
| Cases | 5 | 3 | 1 |  | Homozygous | 0.39 | 2.16 (0.38-12.32) |
| Controls | 18 | 5 | 1 |  | Dominant | 0.40 | 2.40 (0.48-11.97) |
|  |  |  |  |  | Recessive | 0.48 | 2.88 (0.16-51.534) |
|  |  |  |  |  |  |  |  |
| rs17179108 | C/C | C/T | T/T | 0.52 | Heterozygous | 0.39 | 2.16 (0.38-12.32) |
| Cases | 5 | 3 | 1 |  | Homozygous | 0.43 | 3.60 (0.19-68.34) |
| Controls | 18 | 5 | 1 |  | Dominant | 0.40 | 2.40 (0.48-11.97) |
|  |  |  |  |  | Recessive | 0.48 | 2.88 (0.16-51.53) |
|  |  |  |  |  |  |  |  |
| rs1063320 | G/G | C/G | C/C | 0.93 | Heterozygous | 1.00 | 1.13 (0.20-6.43) |
| Cases | 4 | 3 | 2 |  | Homozygous | 1.00 | 1.50 (0.20-11.54) |
| Controls | 12 | 8 | 4 |  | Dominant | 1.00 | 1.25 (0.27-5.83) |
|  |  |  |  |  | Recessive | 1.00 | 1.43 (0.21-9.58) |
|  |  |  |  |  |  |  |  |
| rs9380142 | A/A | A/G | G/G | 0.87 | Heterozygous | 1.00 | 1.39 (0.24-8.07) |
| Cases | 4 | 3 | 2 |  | Homozygous | 0.63 | 1.63 (0.21-12.42) |
| Controls | 13 | 7 | 4 |  | Dominant | 0.71 | 1.48 (0.32-6.90) |
|  |  |  |  |  | Recessive | 1.00 | 1.43 (0.21-9.58) |
|  |  |  |  |  |  |  |  |
| rs1610696 | C/C | C/G | G/G | 1.00 | Heterozygous | 1.00 | 1.38 (0.11-17.32) |
| Cases | 8 | 1 | 0 |  | Homozygous | NA | NA |
| Controls | 22 | 2 | 0 |  | Dominant | 1.00 | 1.38 (0.11-17.32) |
|  |  |  |  |  | Recessive | NA | NA |
|  |  |  |  |  |  |  |  |
| rs1233331 | G/G | A/G | A/A | NA | Heterozygous | NA | NA |
| Cases | 9 | 0 | 0 |  | Homozygous | NA | NA |
| Controls | 24 | 0 | 0 |  | Dominant | NA | NA |
|  |  |  |  |  | Recessive | NA | NA |
|  |  |  |  |  |  |  |  |
| Chronic GVHD |  |  |  |  |  |  |  |
| rs371194629 | del/del | del/ins | ins/ins | 0.05 | Heterozygous | 0.03* | 2.98 (1.10-8.07) |
| Cases | 33 | 43 | 9 |  | Homozygous | 0.25 | 4.36 (0.51-37.48) |
| Controls | 16 | 7 | 1 |  | Dominant | 0.02* | 3.15 (1.21-8.18) |
|  |  |  |  |  | Recessive | 0.45 | 2.72 (0.33-22.65) |
|  |  |  |  |  |  |  |  |
| rs1707 | T/T | C/T | C/C | 0.39 | Heterozygous | 0.39 | 0.27 (0.02-4.55) |
| Cases | 84 | 1 | 0 |  | Homozygous | NA | NA |
| Controls | 23 | 1 | 0 |  | Dominant | 0.39 | 0.27 (0.02-4.55) |
|  |  |  |  |  | Recessive | NA | NA |
|  |  |  |  |  |  |  |  |
| rs1710 | C/C | C/G | G/G | 0.20 | Heterozygous | 0.09 | 2.38 (0.87-6.52) |
| Cases | 29 | 46 | 10 |  | Homozygous | 1.00 | 1.03 (0.27-3.95) |
| Controls | 12 | 8 | 4 |  | Dominant | 0.16 | 1.93 (0.77-4.83) |
|  |  |  |  |  | Recessive | 0.50 | 0.67 (0.19-2.35) |
|  |  |  |  |  |  |  |  |
| rs17179101 | C/C | A/C | A/A | 0.22 | Heterozygous | 0.08 | 2.55 (0.86-7.54) |
| Cases | 48 | 34 | 3 |  | Homozygous | 1.00 | 1.13 (0.11-11.53) |
| Controls | 18 | 5 | 1 |  | Dominant | 0.10 | 2.31 (0.84-6.40) |
|  |  |  |  |  | Recessive | 1.00 | 0.84 (0.08-8.48) |
|  |  |  |  |  |  |  |  |
| rs17179108 | C/C | C/T | T/T | 0.22 | Heterozygous | 0.08 | 2.55 (0.86-7.54) |
| Cases | 48 | 34 | 3 |  | Homozygous | 1.00 | 1.13 (0.11-11.53) |
| Controls | 18 | 5 | 1 |  | Dominant | 0.10 | 2.31 (0.84-6.40) |
|  |  |  |  |  | Recessive | 1.00 | 0.84 (0.08-8.48) |
|  |  |  |  |  |  |  |  |
| rs1063320 | G/G | C/G | C/C | 0.20 | Heterozygous | 0.09 | 2.38 (0.87-6.52) |
| Cases | 29 | 46 | 10 |  | Homozygous | 1.00 | 1.03 (0.27-3.95) |
| Controls | 12 | 8 | 4 |  | Dominant | 0.16 | 1.93 (0.77-4.83) |
|  |  |  |  |  | Recessive | 0.50 | 0.67 (0.19-2.35) |
|  |  |  |  |  |  |  |  |
| rs9380142 | A/A | A/G | G/G | 0.08 | Heterozygous | 0.03* | 3.01 (1.08-8.42) |
| Cases | 29 | 47 | 9 |  | Homozygous | 1.00 | 1.01 (0.26-3.88) |
| Controls | 13 | 7 | 4 |  | Dominant | 0.08 | 2.28 (0.91-5.73) |
|  |  |  |  |  | Recessive | 0.48 | 0.59 (0.17-2.12) |
|  |  |  |  |  |  |  |  |
| rs1610696 | C/C | C/G | G/G | 0.08 | Heterozygous | 0.08 | 3.61 (0.78-16.66) |
| Cases | 64 | 21 | 0 |  | Homozygous | NA | NA |
| Controls | 22 | 2 | 0 |  | Dominant | 0.08 | 3.61 (0.78-16.66) |
|  |  |  |  |  | Recessive | NA | NA |
|  |  |  |  |  |  |  |  |
| rs1233331 | G/G | A/G | A/A | NA | Heterozygous | NA | NA |
| Cases | 85 | 0 | 0 |  | Homozygous | NA | NA |
| Controls | 24 | 0 | 0 |  | Dominant | NA | NA |
|  |  |  |  |  | Recessive | NA | NA |
|  |  |  |  |  |  |  |  |
| Any form of GVHD |  |  |  |  |  |  |  |
| rs371194629 | del/del | del/ins | ins/ins | 0.12 | Heterozygous | 0.07 | 2.40 (0.92-6.22) |
| Cases | 62 | 65 | 13 |  | Homozygous | 0.45 | 3.36 (0.41-27.59) |
| Controls | 16 | 7 | 1 |  | Dominant | 0.04* | 2.52 (1.01-6.26) |
|  |  |  |  |  | Recessive | 0.70 | 2.35 (0.29-18.88) |
|  |  |  |  |  |  |  |  |
| rs1707 | T/T | C/T | C/C | 0.27 | Heterozygous | 0.27 | 0.17 (0.01-2.74) |
| Cases | 139 | 1 | 0 |  | Homozygous | NA | NA |
| Controls | 23 | 1 | 0 |  | Dominant | 0.27 | 0.17 (0.01-2.74) |
|  |  |  |  |  | Recessive | NA | NA |
|  |  |  |  |  |  |  |  |
| rs1710 | C/C | C/G | G/G | 0.29 | Heterozygous | 0.13 | 2.09 (0.80-5.48) |
| Cases | 51 | 71 | 18 |  | Homozygous | 1.00 | 1.06 (0.30-3.71) |
| Controls | 12 | 8 | 4 |  | Dominant | 0.21 | 1.75 (0.73-4.17) |
|  |  |  |  |  | Recessive | 0.54 | 0.74 (0.23-2.41) |
|  |  |  |  |  |  |  |  |
| rs17179101 | C/C | A/C | A/A | 0.29 | Heterozygous | 1.00 | 1.32 (0.15-11.62) |
| Cases | 82 | 52 | 6 |  | Homozygous | 0.12 | 2.28 (0.80-6.52) |
| Controls | 18 | 5 | 1 ( |  | Dominant | 0.13 | 2.12 (0.79-5.67) |
|  |  |  |  |  | Recessive | 1.00 | 1.03 (0.12-8.95) |
|  |  |  |  |  |  |  |  |
| rs17179108 | C/C | C/T | T/T | 0.24 | Heterozygous | 0.09 | 2.43 (0.85-6.94) |
| Cases | 80 | 54 | 6 |  | Homozygous | 1.00 | 1.35 (0.15-11.9) |
| Controls | 18 | 5 | 1 |  | Dominant | 0.10 | 2.25 (0.84-6.01) |
|  |  |  |  |  | Recessive | 1.00 | 1.03 (0.12-8.95) |
|  |  |  |  |  |  |  |  |
| rs1063320 | G/G | C/G | C/C | 0.29 | Heterozygous | 0.13 | 2.09 (0.80-5.48) |
| Cases | 51 | 71 | 18 |  | Homozygous | 1.00 | 1.06 (0.30-3.71) |
| Controls | 12 | 8 | 4 |  | Dominant | 0.21 | 1.75 (0.73-4.17) |
|  |  |  |  |  | Recessive | 0.54 | 0.74 (0.23-2.41) |
|  |  |  |  |  |  |  |  |
| rs9380142 | A/A | A/G | G/G | 0.13 | Heterozygous | 0.05* | 2.62 (0.98-7.03) |
| Cases | 51 | 72 | 17 |  | Homozygous | 1.00 | 1.08 (0.31-3.77) |
| Controls | 13 | 7 | 4 |  | Dominant | 0.10 | 2.06 (0.86-4.94) |
|  |  |  |  |  | Recessive | 0.52 | 0.69 (0.21-2.27) |
|  |  |  |  |  |  |  |  |
| rs1610696 | C/C | C/G | G/G | 0.37 | Heterozygous | 0.37 | 2.39 (0.53-10.83) |
| Cases | 115 | 25 | 0 |  | Homozygous | NA | NA |
| Controls | 22 | 2 | 0 |  | Dominant | 0.37 | 2.39 (0.53-10.83) |
|  |  |  |  |  | Recessive | NA | NA |
|  |  |  |  |  |  |  |  |
| rs1233331 | G/G | A/G | A/A | NA | Heterozygous | NA | NA |
| Cases | 140 | 0 | 0 |  | Homozygous | NA | NA |
| Controls | 24 | 0 | 0 |  | Dominant | NA | NA |
|  |  |  |  |  | Recessive | NA | NA |
| ^a^Cases: patients with the event; Controls: patients are alive, without relapse, or without GVHD; *:p < 0.05; NA: not applicable. | | | | | | | |

**Supplementary Table S2.** Association study of the genetic variants in 5’-URR of HLA-G with various adverse outcomes post-HSCT.

| **SNP** | **No. of patients (%)** | | | **Additive p** | **Model** | **Logistic regression p** | **OR (95 % CI)** |
| --- | --- | --- | --- | --- | --- | --- | --- |
| Mortality |  |  |  |  |  |  |  |
| rs1736936 | A/A | A/G | G/G | 0.99 | Heterozygous | 0.98 | 1.01 (0.51-1.99) |
| Cases^a^ | 26 | 32 | 10 |  | Homozygous | 0.90 | 1.07 (0.41-2.80) |
| Controls^a^ | 36 | 44 | 13 |  | Dominant | 0.95 | 1.02 (0.54-1.94) |
|  |  |  |  |  | Recessive | 0.90 | 1.06 (0.44-2.59) |
|  |  |  |  |  |  |  |  |
| rs1736935 | G/G | A/G | A/A | 0.95 | Heterozygous | 0.96 | 1.02 (0.52-2.00) |
| Cases | 26 | 32 | 10 |  | Homozygous | 0.77 | 0.87 (0.33-2.31) |
| Controls | 36 | 45 | 12 |  | Dominant | 0.95 | 0.98 (0.52-1.86) |
|  |  |  |  |  | Recessive | 0.74 | 1.86 (0.35-2.12.) |
|  |  |  |  |  |  |  |  |
| rs3823321 | G/G | A/G | A/A | 0.30 | Heterozygous | 0.27 | 1.47 (0.75-2.89) |
| Cases | 31 | 30 | 7 |  | Homozygous | 0.50 | 0.71 (0.26-1.93) |
| Controls | 47 | 31 | 15 |  | Dominant | 0.54 | 1.22 (0.65-2.28) |
|  |  |  |  |  | Recessive | 0.29 | 0.60 (0.23-1.56) |
|  |  |  |  |  |  |  |  |
| rs1736934 | A/A | A/T | T/T | 1.00 | Heterozygous | 0.93 | 0.97 (0.50-1.87) |
| Cases | 34 | 28 | 6 |  | Homozygous | 0.98 | 1.02 (0.32-3.20) |
| Controls | 46 | 39 | 8 |  | Dominant | 0.95 | 0.98 (0.52-1.83) |
|  |  |  |  |  | Recessive | 0.96 | 1.03 (0.34-3.11) |
|  |  |  |  |  |  |  |  |
| rs1632947 | A/A | A/G | G/G | 0.99 | Heterozygous | 0.98 | 1.01 (0.51-1.99) |
| Cases | 26 | 32 | 10 |  | Homozygous | 0.90 | 1.07 (0.41-2.80) |
| Controls | 36 | 44 | 13 |  | Dominant | 0.95 | 1.02 (0.54-1.94) |
|  |  |  |  |  | Recessive | 0.90 | 1.06 (0.44-2.59) |
|  |  |  |  |  |  |  |  |
| rs1632946 | T/T | C/T | C/C | 0.99 | Heterozygous | 0.98 | 1.01 (0.51-1.99) |
| Cases | 26 | 32 | 10 |  | Homozygous | 0.90 | 1.07 (0.41-2.80) |
| Controls | 36 | 44 | 13 |  | Dominant | 0.95 | 1.02 (0.54-1.94) |
|  |  |  |  |  | Recessive | 0.90 | 1.06 (0.44-2.59) |
|  |  |  |  |  |  |  |  |
| rs1233334 | C/C | C/G | C/T | 0.68 | Heterozygous | 1.00 | 1.36 (0.08-22.11) |
| Cases | 67 | 1 | 0 |  | Heterozygous | 1.00 | NA |
| Controls | 91 | 1 | 1 |  |  |  |  |
|  |  |  |  |  |  |  |  |
| rs2249863 | G/G | T/G | T/T | 0.99 | Heterozygous | 0.98 | 1.01 (0.51-1.99) |
| Cases | 26 | 32 | 10 |  | Homozygous | 0.90 | 1.07 (0.41-2.80) |
| Controls | 36 | 44 | 13 |  | Dominant | 0.95 | 1.02 (0.54-1.94) |
|  |  |  |  |  | Recessive | 0.90 | 1.06 (0.44-2.59) |
|  |  |  |  |  |  |  |  |
| rs2735022 | G/G | A/G | A/A | 0.99 | Heterozygous | 0.98 | 1.01 (0.51-1.99) |
| Cases | 26 | 32 | 10 |  | Homozygous | 0.90 | 1.07 (0.41-2.80) |
| Controls | 36 | 44 | 13 |  | Dominant | 0.95 | 1.02 (0.54-1.94) |
|  |  |  |  |  | Recessive | 0.90 | 1.06 (0.44-2.57) |
|  |  |  |  |  |  |  |  |
| rs35674592 | T/T | T/G | G/G | 0.99 | Heterozygous | 0.98 | 1.01 (0.51-1.99) |
| Cases | 26 | 32 | 10 |  | Homozygous | 0.90 | 1.07 (0.41-2.80) |
| Controls | 36 | 44 | 13 |  | Dominant | 0.95 | 1.02 (0.54-1.94) |
|  |  |  |  |  | Recessive | 0.90 | 1.06 (0.44-2.59) |
|  |  |  |  |  |  |  |  |
| rs1632944 | A/A | A/G | G/G | 0.99 | Heterozygous | 0.98 | 1.01 (0.51-1.99) |
| Cases | 26 | 32 | 10 |  | Homozygous | 0.90 | 1.07 (0.41-2.80) |
| Controls | 36 | 44 | 13 |  | Dominant | 0.95 | 1.02 (0.54-1.94) |
|  |  |  |  |  | Recessive | 0.90 | 1.06 (0.44-2.59) |
|  |  |  |  |  |  |  |  |
| rs1736933 | C/C | A/C | A/A | 0.92 | Heterozygous | 0.90 | 1.05 (0.53-2.08) |
| Cases | 25 | 32 | 11 |  | Homozygous | 0.68 | 1.22 (0.47-3.16) |
| Controls | 36 | 44 | 13 |  | Dominant | 0.80 | 1.09 (0.57-2.08) |
|  |  |  |  |  | Recessive | 0.70 | 1.19 (0.50-2.84) |
|  |  |  |  |  |  |  |  |
| rs1736932 | G/G | C/G | C/C | 0.87 | Heterozygous | 0.97 | 0.99 (0.50-1.94) |
| Cases | 26 | 32 | 10 |  | Homozygous | 0.61 | 0.77 (0.29-2.09) |
| Controls | 37 | 45 | 11 |  | Dominant | 0.84 | 0.94 (0.49-1.78) |
|  |  |  |  |  | Recessive | 0.59 | 1.29 (0.51-3.23) |
|  |  |  |  |  |  |  |  |
| rs17875394 | G/G | A/G | A/A | 0.64 | Heterozygous | 0.53 | 1.31 (0.57-3.00) |
| Cases | 54 | 13 | 1 |  | Homozygous | 0.64 | 0.47 (0.05-4.63) |
| Controls | 76 | 14 | 3 |  | Dominant | 0.71 | 1.16 (0.53-2.55) |
|  |  |  |  |  | Recessive | 0.64 | 0.45 (0.05-4.40) |
|  |  |  |  |  |  |  |  |
| rs1632943 | A/A | A/C | C/C | 0.84 | Heterozygous | 0.95 | 1.02 (0.52-2.03) |
| Cases | 25 | 32 | 11 |  | Homozygous | 0.57 | 1.32 (0.50-3.46) |
| Controls | 36 | 45 | 12 |  | Dominant | 0.80 | 1.09 (0.57-2.07) |
|  |  |  |  |  | Recessive | 0.56 | 1.30 (0.54-3.16) |
|  |  |  |  |  |  |  |  |
| rs1233333 | A/A | A/G | G/G | 0.99 | Heterozygous | 0.98 | 1.01 (0.51-1.99) |
| Cases | 26 | 32 | 10 |  | Homozygous | 0.90 | 1.07 (0.41-2.80) |
| Controls | 36 | 44 | 13 |  | Dominant | 0.95 | 1.02 (0.54-1.94) |
|  |  |  |  |  | Recessive | 0.90 | 1.06 (0.44-2.59) |
|  |  |  |  |  |  |  |  |
| Relapse |  |  |  |  |  |  |  |
| rs1736936 | A/A | A/G | G/G | 0.07 | Heterozygous | 0.09 | 0.55 (0.28-1.09) |
| Cases | 22 | 38 | 6 |  | Homozygous | 0.41 | 1.56 (0.54-4.53) |
| Controls | 40 | 38 | 17 |  | Dominant | 0.26 | 0.69 (0.36-1.32) |
|  |  |  |  |  | Recessive | 0.12 | 2.18 (0.81-5.86) |
|  |  |  |  |  |  |  |  |
| rs1736935 | G/G | A/G | A/A | 0.10 | Heterozygous | 0.10 | 1.77 (0.89-3.52) |
| Cases | 22 | 38 | 6 |  | Homozygous | 0.48 | 0.68 (0.23-1.99) |
| Controls | 40 | 39 | 17 |  | Dominant | 0.26 | 1.45 0.76-2.80) |
|  |  |  |  |  | Recessive | 0.16 | 0.49 (0.18-1.34) |
|  |  |  |  |  |  |  |  |
| rs3823321 | G/G | A/G | A/A | 0.77 | Heterozygous | 0.59 | 0.83 (0.42-1.64) |
| Cases | 31 | 27 | 8 |  | Homozygous | 0.77 | 1.15 (0.43-3.08) |
| Controls | 47 | 34 | 14 |  | Dominant | 0.76 | 0.91 (0.49-1.70) |
|  |  |  |  |  | Recessive | 0.64 | 1.26 (0.50-3.18) |
|  |  |  |  |  |  |  |  |
| rs1736934 | A/A | A/T | T/T | 0.11 | Heterozygous | 0.15 | 0.62 (0.32-1.20) |
| Cases | 30 | 33 | 3 |  | Homozygous | 0.37 | 2.20 (0.57-8.53) |
| Controls | 50 | 34 | 11 |  | Dominant | 0.37 | 0.75 (0.40-1.41) |
|  |  |  |  |  | Recessive | 0.12 | 2.75 (0.74-10.28) |
|  |  |  |  |  |  |  |  |
| rs1632947 | A/A | A/G | G/G | 0.07 | Heterozygous | 0.09 | 0.55 (0.28-1.09) |
| Cases | 22 | 38 | 6 |  | Homozygous | 0.41 | 1.56 (0.54-4.53) |
| Controls | 40 | 38 | 17 |  | Dominant | 0.26 | 0.69 (0.36-1.33) |
|  |  |  |  |  | Recessive | 0.12 | 2.18 (0.8-5.86) |
|  |  |  |  |  |  |  |  |
| rs1632946 | T/T | C/T | C/C | 0.07 | Heterozygous | 0.09 | 0.55 (0.28-1.09) |
| Cases | 22 | 38 | 6 |  | Homozygous | 0.41 | 1.56 (0.54-4.53) |
| Controls | 40 | 38 | 17 |  | Dominant | 0.26 | 0.69 (0.36-1.32) |
|  |  |  |  |  | Recessive | 0.12 | 2.18 (0.81-5.86) |
|  |  |  |  |  |  |  |  |
| rs1233334 | C/C | C/G | C/T | 0.35 | Heterozygous | 0.51 | NA |
| Cases | 66 | 0 | 0 |  | Heterozygous | 1.00 | NA |
| Controls | 92 | 2 | 1 |  |  |  |  |
|  |  |  |  |  |  |  |  |
| rs2249863 | G/G | T/G | T/T | 0.07 | Heterozygous | 0.09 | 0.55 (0.28-1.09) |
| Cases | 22 | 38 | 6 |  | Homozygous | 0.41 | 1.56 (0.54-4.53) |
| Controls | 40 | 38 | 17 |  | Dominant | 0.26 | 0.69 (0.36-1.32) |
|  |  |  |  |  | Recessive | 0.12 | 2.18 (0.80-5.86) |
|  |  |  |  |  |  |  |  |
| rs2735022 | G/G | A/G | A/A | 0.07 | Heterozygous | 0.09 | 0.55 (0.28-1.10) |
| Cases | 22 | 38 | 6 |  | Homozygous | 0.41 | 1.56 (0.54-4.53) |
| Controls | 40 | 38 | 17 |  | Dominant | 0.26 | 0.69 (0.36-1.33) |
|  |  |  |  |  | Recessive | 0.12 | 2.18 (0.80-5.86) |
|  |  |  |  |  |  |  |  |
| rs35674592 | T/T | T/G | G/G | 0.07 | Heterozygous | 0.09 | 0.55 (0.28-1.10) |
| Cases | 22 | 38 | 6 |  | Homozygous | 0.41 | 1.56 (0.54-4.53) |
| Controls | 40 | 38 | 17 |  | Dominant | 0.26 | 0.69 (0.36-1.33) |
|  |  |  |  |  | Recessive | 0.12 | 2.18 (0.80-5.86) |
|  |  |  |  |  |  |  |  |
| rs1632944 | A/A | A/G | G/G | 0.07 | Heterozygous | 0.09 | 0.55 (0.28-1.10) |
| Cases | 22 | 38 | 6 |  | Homozygous | 0.41 | 1.56 (0.54-4.53) |
| Controls | 40 | 38 | 17 |  | Dominant | 0.26 | 0.69 (0.36-1.33) |
|  |  |  |  |  | Recessive | 0.12 | 2.18 (0.80-5.86) |
|  |  |  |  |  |  |  |  |
| rs1736933 | C/C | A/C | A/A | 0.06 | Heterozygous | 0.10 | 0.56 (0.28-1.12) |
| Cases | 22 | 38 | 6 |  | Homozygous | 0.33 | 1.69 (0.59-4.89) |
| Controls | 39 | 38 | 18 |  | Dominant | 0.32 | 0.72 (0.37-1.38) |
|  |  |  |  |  | Recessive | 0.08 | 2.34 (0.87-6.25) |
|  |  |  |  |  |  |  |  |
| rs1736932 | G/G | C/G | C/C | 0.10 | Heterozygous | 0.09 | 1.82 (0.92-3.60) |
| Cases | 22 | 38 | 6 |  | Homozygous | 0.59 | 0.75 (0.25-2.19) |
| Controls | 41 | 39 | 15 |  | Dominant | 0.21 | 1.52 (0.79-2.92) |
|  |  |  |  |  | Recessive | 0.21 | 1.88 (0.69-5.12) |
|  |  |  |  |  |  |  |  |
| rs17875394 | G/G | A/G | A/A | 0.39 | Heterozygous | 0.23 | 0.60 (0.26-1.38) |
| Cases | 51 | 14 | 1 |  | Homozygous | 1.00 | 1.94 (0.20-19.13) |
| Controls | 79 | 13 | 3 |  | Dominant | 0.35 | 0.69 (0.31-1.51) |
|  |  |  |  |  | Recessive | 0.65 | 2.12 (0.22-20.83) |
|  |  |  |  |  |  |  |  |
| rs1632943 | A/A | A/C | C/C | 0.11 | Heterozygous | 0.08 | 0.54 (0.27-1.08) |
| Cases | 21 | 38 | 7 |  | Homozygous | 0.73 | 1.20 (0.43-3.37) |
| Controls | 40 | 39 | 16 |  | Dominant | 0.19 | 0.64 (0.33-1.24) |
|  |  |  |  |  | Recessive | 0.27 | 1.71 (0.66-4.41) |
|  |  |  |  |  |  |  |  |
| rs1233333 | A/A | A/G | G/G | 0.07 | Heterozygous | 0.09 | 0.55 (0.28-1.09) |
| Cases | 40 | 38 | 17 |  | Homozygous | 0.41 | 1.56 (0.54-4.53) |
| Controls | 22 | 38 | 6 |  | Dominant | 0.26 | 0.69 (0.36-1.32) |
|  |  |  |  |  | Recessive | 0.12 | 2.18 (0.81-5.86) |
|  |  |  |  |  |  |  |  |
| Grades I–II |  |  |  |  |  |  |  |
| rs1736936 | A/A | A/G | G/G | 0.60 | Heterozygous | 0.32 | 1.75 (0.59-5.23) |
| Cases | 18 | 21 | 7 |  | Homozygous | 1.00 | 1.17 (0.29-4.87) |
| Controls | 12 | 8 | 4 |  | Dominant | 0.38 | 1.56 (0.58-4.21) |
|  |  |  |  |  | Recessive | 1.00 | 0.90 (0.24-3.43) |
|  |  |  |  |  |  |  |  |
| rs1736935 | G/G | A/G | A/A | 0.51 | Heterozygous | 0.27 | 1.55 (0.18-1.62) |
| Cases | 18 | 22 | 6 |  | Homozygous | 1.00 | 1.00 (0.23-4.31) |
| Controls | 12 | 8 | 4 |  | Dominant | 0.38 | 0.64 (0.24-1.74) |
|  |  |  |  |  | Recessive | 0.73 | 0.75 (0.19-2.96) |
|  |  |  |  |  |  |  |  |
| rs3823321 | G/G | A/G | A/A | 0.19 | Heterozygous | 0.61 | 1.35 (0.42-4.36) |
| Cases | 19 | 20 | 7 |  | Homozygous | 0.18 | 0.41 (0.11-1.50) |
| Controls | 9 | 7 | 8 |  | Dominant | 0.76 | 0.85 (0.31-2.35) |
|  |  |  |  |  | Recessive | 0.08 | 0.36 (0.11-1.16) |
|  |  |  |  |  |  |  |  |
| rs1736934 | A/A | A/T | T/T | 0.70 | Heterozygous | 0.46 | 1.50 (0.51-4.39) |
| Cases | 26 | 17 | 3 |  | Homozygous | 1.00 | 1.85 (0.18-19.31) |
| Controls | 16 | 7 | 1 |  | Dominant | 0.41 | 1.54 (0.55-4.31) |
|  |  |  |  |  | Recessive | 1.00 | 1.61 (0.16-16.31) |
|  |  |  |  |  |  |  |  |
| rs1632947 | A/A | A/G | G/G | 0.60 | Heterozygous | 0.31 | 1.75 (0.59-5.23) |
| Cases | 18 | 21 | 7 |  | Homozygous | 1.00 | 1.17 (0.28-4.87) |
| Controls | 12 | 8 | 4 |  | Dominant | 0.38 | 1.56 (0.58-4.21) |
|  |  |  |  |  | Recessive | 1.00 | 0.88 (0.24-3.43) |
|  |  |  |  |  |  |  |  |
| rs1632946 | T/T | C/T | C/C | 0.60 | Heterozygous | 0.31 | 0.57 (0.19-1.71) |
| Cases | 18 | 21 | 7 |  | Homozygous | 1.00 | 0.86 (0.21-3.58) |
| Controls | 12 | 8 | 4 |  | Dominant | 0.38 | 0.64 (0.24-1.74) |
|  |  |  |  |  | Recessive | 1.00 | 1.11 (0.29-4.26) |
|  |  |  |  |  |  |  |  |
| rs1233334 | C/C | C/G | C/T | 0.30 | Heterozygous | 0.35 | NA |
| Cases | 45 | 0 | 1 |  | Heterozygous | 1.00 | NA |
| Controls | 23 | 1 | 0 |  |  |  |  |
|  |  |  |  |  |  |  |  |
| rs2249863 | G/G | T/G | T/T | 0.60 | Heterozygous | 0.31 | 1.75 (0.59-5.23) |
| Cases | 18 | 21 | 7 |  | Homozygous | 1.00 | 1.17 (0.28-4.87) |
| Controls | 12 | 8 | 4 |  | Dominant | 0.38 | 1.56 (0.58-4.21) |
|  |  |  |  |  | Recessive | 1.00 | 0.88 (0.24-3.43) |
|  |  |  |  |  |  |  |  |
| rs2735022 | G/G | A/G | A/A | 0.60 | Heterozygous | 0.31 | 1.75 (0.59-5.23) |
| Cases | 18 | 21 | 7 |  | Homozygous | 1.00 | 1.17 (0.28-4.87) |
| Controls | 12 | 8 | 4 |  | Dominant | 0.38 | 1.56 (0.58-4.21) |
|  |  |  |  |  | Recessive | 1.00 | 0.88 (0.24-3.43) |
|  |  |  |  |  |  |  |  |
| rs35674592 | T/T | T/G | G/G | 0.60 | Heterozygous | 0.31 | 1.75 (0.59-5.23) |
| Cases | 18 | 21 | 7 |  | Homozygous | 1.00 | 1.17 (0.28-4.87) |
| Controls | 12 | 8 | 4 |  | Dominant | 0.38 | 1.56 (0.58-4.21) |
|  |  |  |  |  | Recessive | 1.00 | 0.88 (0.24-3.43) |
|  |  |  |  |  |  |  |  |
| rs1632944 | A/A | A/G | G/G | 0.60 | Heterozygous | 0.31 | 1.75 (0.59-5.23) |
| Cases | 18 | 21 | 7 |  | Homozygous | 1.00 | 1.17 (0.28-4.87) |
| Controls | 12 | 8 | 4 |  | Dominant | 0.38 | 1.56 (0.58-4.21) |
|  |  |  |  |  | Recessive | 1.00 | 0.88 (0.24-3.43) |
|  |  |  |  |  |  |  |  |
| rs1736933 | C/C | A/C | A/A | 0.60 | Heterozygous | 0.31 | 1.75 (0.59-5.23) |
| Cases | 18 | 21 | 7 |  | Homozygous | 1.00 | 1.17 (0.28-4.87) |
| Controls | 12 | 8 | 4 |  | Dominant | 0.38 | 1.56 (0.58-4.21) |
|  |  |  |  |  | Recessive | 1.00 | 0.88 (0.24-3.43) |
|  |  |  |  |  |  |  |  |
| rs1736932 | G/G | C/G | C/C | 0.48 | Heterozygous | 0.32 | 0.58 (0.19-1.70) |
| Cases | 19 | 22 | 5 |  | Homozygous | 1.00 | 1.27 (0.28-5.68) |
| Controls | 12 | 8 | 4 |  | Dominant | 0.71 | 0.49 (0.70-0.26) |
|  |  |  |  |  | Recessive | 0.48 | 0.61 (0.15-2.52) |
|  |  |  |  |  |  |  |  |
| rs17875394 | G/G | A/G | A/A | 0.54 | Heterozygous | 1.00 | 1.29 (0.35-4.72) |
| Cases | 35 | 9 | 2 |  | Homozygous | 0.54 | NA |
| Controls | 20 | 4 | 0 |  | Dominant | 0.48 | 1.57 (0.44-5.59) |
|  |  |  |  |  | Recessive | 0.54 | NA |
|  |  |  |  |  |  |  |  |
| rs1632943 | A/A | A/C | C/C | 0.51 | Heterozygous | 0.27 | 1.83 (0.62-5.45) |
| Cases | 18 | 22 | 6 |  | Homozygous | 1.00 | 1.00 (0.23-4.31) |
| Controls | 12 | 8 | 4 |  | Dominant | 0.38 | 1.56 (0.58-4.21) |
|  |  |  |  |  | Recessive | 0.73 | 0.75 (0.19-2.96) |
|  |  |  |  |  |  |  |  |
| rs1233333 | A/A | A/G | G/G | 0.60 | Heterozygous | 0.31 | 1.75 (0.59-5.23) |
| Cases | 18 | 21 | 7 |  | Homozygous | 1.00 | 1.17 (0.28-4.87) |
| Controls | 12 | 8 | 4 |  | Dominant | 0.38 | 1.56 (0.58-4.21) |
|  |  |  |  |  | Recessive | 1.00 | 0.90 (0.24-3.43) |
|  |  |  |  |  |  |  |  |
| Grades III–IV |  |  |  |  |  |  |  |
| rs1736936 | A/A | A/G | G/G | 0.93 | Heterozygous | 1.00 | 1.13 (0.20-6.43) |
| Cases | 4 | 3 | 2 |  | Homozygous | 1.00 | 1.50 (0.20-11.54) |
| Controls | 12 | 8 | 4 |  | Dominant | 1.00 | 1.25 (0.27-5.83) |
|  |  |  |  |  | Recessive | 1.00 | 1.43 (0.21-9.58) |
|  |  |  |  |  |  |  |  |
| rs1736935 | G/G | A/G | A/A | 0.93 | Heterozygous | 1.00 | 1.13 (0.20-6.43) |
| Cases | 4 | 3 | 2 |  | Homozygous | 1.00 | 1.50 (0.20-11.54) |
| Controls | 12 | 8 | 4 |  | Dominant | 1.00 | 1.25 (0.27-5.83) |
|  |  |  |  |  | Recessive | 1.00 | 1.43 (0.21-9.58) |
|  |  |  |  |  |  |  |  |
| rs3823321 | G/G | A/G | A/A | 0.42 | Heterozygous | 1.00 | 1.29 (0.23-7.05) |
| Cases | 4 | 4 | 1 |  | Homozygous | 0.36 | 0.28 (0.03-3.07) |
| Controls | 9 | 7 | 8 |  | Dominant | 1.00 | 0.75 (0.16-3.54) |
|  |  |  |  |  | Recessive | 0.38 | 0.25 (0.03-2.36) |
|  |  |  |  |  |  |  |  |
| rs1736934 | A/A | A/T | T/T | 0.71 | Heterozygous | 1.00 | 1.37 (0.25-7.39) |
| Cases | 5 | 3 | 1 |  | Homozygous | 0.46 | 3.20 (0.17-61.02) |
| Controls | 16 | 7 | 1 |  | Dominant | 0.69 | 1.60 (0.34-7.65) |
|  |  |  |  |  | Recessive | 0.48 | 2.88 (0.16-51.53) |
|  |  |  |  |  |  |  |  |
| rs1632947 | A/A | A/G | G/G | 0.93 | Heterozygous | 1.00 | 1.13 (0.20-6.43) |
| Cases | 4 | 3 | 2 |  | Homozygous | 1.00 | 1.50 (0.20-11.54) |
| Controls | 12 | 8 | 4 |  | Dominant | 1.00 | 1.25 (0.27-5.83) |
|  |  |  |  |  | Recessive | 1.00 | 1.43 (0.21-9.58) |
|  |  |  |  |  |  |  |  |
| rs1632946 | T/T | C/T | C/C | 0.93 | Heterozygous | 1.00 | 1.13 (0.20-6.43) |
| Cases | 4 | 3 | 2 |  | Homozygous | 1.00 | 1.50 (0.20-11.54) |
| Controls | 12 | 8 | 4 |  | Dominant | 1.00 | 1.25 (0.27-5.83) |
|  |  |  |  |  | Recessive | 1.00 | 1.43 (0.21-9.58) |
|  |  |  |  |  |  |  |  |
| rs1233334 | C/C | C/G | C/T | 1.00 | Heterozygous | 1.00 | NA |
| Cases | 9 | 0 | 0 |  | Heterozygous | NA | NA |
| Controls | 23 | 1 | 0 |  |  |  |  |
|  |  |  |  |  |  |  |  |
| rs2249863 | G/G | T/G | T/T | 0.93 | Heterozygous | 1.00 | 1.13 (0.20-6.43) |
| Cases | 4 | 3 | 2 |  | Homozygous | 1.00 | 1.50 (0.20-11.54) |
| Controls | 12 | 8 | 4 |  | Dominant | 1.00 | 1.25 (0.27-5.83) |
|  |  |  |  |  | Recessive | 1.00 | 1.43 (0.21-9.58) |
|  |  |  |  |  |  |  |  |
| rs2735022 | G/G | A/G | A/A | 0.93 | Heterozygous | 1.00 | 1.13 (0.20-6.43) |
| Cases | 4 | 3 | 2 |  | Homozygous | 1.00 | 1.50 (0.20-11.54) |
| Controls | 12 | 8 | 4 |  | Dominant | 1.00 | 1.25 (0.27-5.83) |
|  |  |  |  |  | Recessive | 1.00 | 1.43 (0.21-9.58) |
|  |  |  |  |  |  |  |  |
| rs35674592 | T/T | T/G | G/G | 0.93 | Heterozygous | 1.00 | 1.13 (0.20-6.43) |
| Cases | 4 | 3 | 2 |  | Homozygous | 1.00 | 1.50 (0.20-11.54) |
| Controls | 12 | 8 | 4 |  | Dominant | 1.00 | 1.25 (0.27-5.83) |
|  |  |  |  |  | Recessive | 1.00 | 1.43 (0.21-9.58) |
|  |  |  |  |  |  |  |  |
| rs1632944 | A/A | A/G | G/G | 0.93 | Heterozygous | 1.00 | 1.13 (0.20-6.43) |
| Cases | 4 | 3 | 2 |  | Homozygous | 1.00 | 1.50 (0.20-11.54) |
| Controls | 12 | 8 | 4 |  | Dominant | 1.00 | 1.25 (0.27-5.83) |
|  |  |  |  |  | Recessive | 1.00 | 1.43 (0.21-9.58) |
|  |  |  |  |  |  |  |  |
| rs1736933 | C/C | A/C | A/A | 0.53 | Heterozygous | 1.00 | 1.50 (0.24-9.38) |
| Cases | 3 | 3 | 3 |  | Homozygous | 0.33 | 3.00 (0.42-21.30) |
| Controls | 12 | 8 | 4 |  | Dominant | 0.46 | 2.00 (0.40-9.91) |
|  |  |  |  |  | Recessive | 0.36 | 2.50 (0.43-14.43) |
|  |  |  |  |  |  |  |  |
| rs1736932 | G/G | C/G | C/C | 0.93 | Heterozygous | 1.00 | 1.13 (0.20-6.43) |
| Cases | 4 | 3 | 2 |  | Homozygous | 1.00 | 1.50 (0.20-11.54) |
| Controls | 12 | 8 | 4 |  | Dominant | 1.00 | 1.25 (0.27-5.83) |
|  |  |  |  |  | Recessive | 1.00 | 1.43 (0.21-9.58) |
|  |  |  |  |  |  |  |  |
| rs17875394 | G/G | A/G | A/A | 1.00 | Heterozygous | 1.00 | 1.43 (0.21-9.58) |
| Cases | 7 | 2 | 0 |  | Homozygous | NA | NA |
| Controls | 20 | 4 | 0 |  | Dominant | 1.00 | 1.43 (0.21-9.58) |
|  |  |  |  |  | Recessive | NA | NA |
|  |  |  |  |  |  |  |  |
| rs1632943 | A/A | A/C | C/C | 0.93 | Heterozygous | 1.00 | 1.13 (0.20-6.43) |
| Cases | 4 | 3 | 2 |  | Homozygous | 1.00 | 1.50 (0.20-11.54) |
| Controls | 12 | 8 | 4 |  | Dominant | 1.00 | 1.25 (0.27-5.83) |
|  |  |  |  |  | Recessive | 1.00 | 1.43 (0.21-9.58) |
|  |  |  |  |  |  |  |  |
| rs1233333 | A/A | A/G | G/G | 0.93 | Heterozygous | 1.00 | 1.13 (0.20-6.43) |
| Cases | 4 | 3 | 2 |  | Homozygous | 1.00 | 1.50 (0.20-11.54) |
| Controls | 12 | 8 | 4 |  | Dominant | 1.00 | 1.25 (0.27-5.83) |
|  |  |  |  |  | Recessive | 1.00 | 1.43 (0.21-9.58) |
|  |  |  |  |  |  |  |  |
| Chronic GVHD |  |  |  |  |  |  |  |
| rs1736936 | A/A | A/G | G/G | 0.21 | Heterozygous | 0.09 | 2.36 (0.86-6.49) |
| Cases | 28 | 44 | 10 |  | Homozygous | 1.00 | 1.07 (0.28-4.10) |
| Controls | 12 | 8 | 4 |  | Dominant | 0.16 | 1.93 (0.77-4.85) |
|  |  |  |  |  | Recessive | 0.52 | 0.69 (0.20-2.45) |
|  |  |  |  |  |  |  |  |
| rs1736935 | G/G | A/G | A/A | 0.21 | Heterozygous | 0.09 | 2.36 (0.86-6.47) |
| Cases | 28 | 44 | 10 |  | Homozygous | 1.00 | 1.07 (0.28-4.10) |
| Controls | 12 | 8 | 4 |  | Dominant | 0.16 | 1.93 (0.77-4.85) |
|  |  |  |  |  | Recessive | 0.52 | 0.69 (0.20-2.45) |
|  |  |  |  |  |  |  |  |
| rs3823321 | G/G | A/G | A/A | <0.01* | Heterozygous | 0.75 | 0.84 (0.28-2.49) |
| Cases | 46 | 30 | 6 |  | Homozygous | <0.01* | 0.15 (0.04-0.53) |
| Controls | 9 | 7 | 8 |  | Dominant | 0.11 | 0.47 (0.18-1.20) |
|  |  |  |  |  | Recessive | <0.01* | 0.16 (0.05-0.52) |
|  |  |  |  |  |  |  |  |
| rs1736934 | A/A | A/T | T/T | 0.07 | Heterozygous | 0.04* | 2.77 (1.02-7.54) |
| Cases | 33 | 40 | 9 |  | Homozygous | 0.25 | 4.36 (0.51-37.48) |
| Controls | 16 | 7 | 1 |  | Dominant | 0.02* | 2.97 (1.14-7.73) |
|  |  |  |  |  | Recessive | 0.45 | 2.84 (0.34-23.59) |
|  |  |  |  |  |  |  |  |
| rs1632947 | A/A | A/G | G/G | 0.21 | Heterozygous | 0.09 | 2.36 (0.86-6.49) |
| Cases | 28 | 44 | 10 |  | Homozygous | 1.00 | 1.07 (0.28-4.10) |
| Controls | 12 | 8 | 4 |  | Dominant | 0.16 | 1.93 (0.77-4.85) |
|  |  |  |  |  | Recessive | 0.52 | 0.70 (0.20-2.45) |
|  |  |  |  |  |  |  |  |
| rs1632946 | T/T | C/T | C/C | 0.21 | Heterozygous | 0.09 | 2.36 (0.86-6.49) |
| Cases | 28 | 44 | 10 |  | Homozygous | 1.00 | 1.07 (0.28-4.10) |
| Controls | 12 | 8 | 4 |  | Dominant | 0.16 | 1.93 (0.77-4.85) |
|  |  |  |  |  | Recessive | 0.52 | 0.70 (0.20-2.45) |
|  |  |  |  |  |  |  |  |
| rs1233334 | C/C | C/G | C/T | 0.40 | Heterozygous | 0.40 | 0.28 (0.02-4.77) |
| Cases | 81 | 1 | 0 |  | Heterozygous | NA | NA |
| Controls | 23 | 1 | 0 |  |  |  |  |
|  |  |  |  |  |  |  |  |
| rs2249863 | G/G | T/G | T/T | 0.21 | Heterozygous | 0.09 | 2.36 (0.86-6.49) |
| Cases | 28 | 44 | 10 |  | Homozygous | 1.00 | 1.07 (0.28-4.10) |
| Controls | 12 | 8 | 4 |  | Dominant | 0.16 | 1.93 (0.77-4.85) |
|  |  |  |  |  | Recessive | 0.52 | 0.70 (0.20-2.45) |
|  |  |  |  |  |  |  |  |
| rs2735022 | G/G | A/G | A/A | 0.21 | Heterozygous | 0.09 | 2.36 (0.86-6.49) |
| Cases | 28 | 44 | 10 |  | Homozygous | 1.00 | 1.07 (0.28-4.10) |
| Controls | 12 | 8 | 4 |  | Dominant | 0.16 | 1.93 (0.77-4.85) |
|  |  |  |  |  | Recessive | 0.52 | 0.70 (0.20-2.45) |
|  |  |  |  |  |  |  |  |
| rs35674592 | T/T | T/G | G/G | 0.21 | Heterozygous | 0.09 | 2.36 (0.86-6.49) |
| Cases | 28 | 44 | 10 |  | Homozygous | 1.00 | 1.07 (0.28-4.10) |
| Controls | 12 | 8 | 4 |  | Dominant | 0.16 | 1.93 (0.77-4.85) |
|  |  |  |  |  | Recessive | 0.52 | 0.69 (0.20-2.45) |
|  |  |  |  |  |  |  |  |
| rs1632944 | A/A | A/G | G/G | 0.21 | Heterozygous | 0.09 | 2.36 (0.86-6.49) |
| Cases | 28 | 44 | 10 |  | Homozygous | 1.00 | 1.07 (0.28-4.10) |
| Controls | 12 | 8 | 4 |  | Dominant | 0.16 | 1.93 (0.77-4.85) |
|  |  |  |  |  | Recessive | 0.52 | 0.69 (0.20-2.45) |
|  |  |  |  |  |  |  |  |
| rs1736933 | C/C | A/C | A/A | 0.21 | Heterozygous | 0.09 | 2.36 (0.86-6.49) |
| Cases | 28 | 44 | 10 |  | Homozygous | 1.00 | 1.07 (0.28-4.10) |
| Controls | 12 | 8 | 4 |  | Dominant | 0.16 | 1.93 (0.77-4.85) |
|  |  |  |  |  | Recessive | 0.52 | 0.69 (0.20-2.45) |
|  |  |  |  |  |  |  |  |
| rs1736932 | G/G | C/G | C/C | 0.21 | Heterozygous | 0.09 | 2.36 (0.86-6.49) |
| Cases | 28 | 44 | 10 |  | Homozygous | 1.00 | 1.07 (0.28-4.10) |
| Controls | 12 | 8 | 4 |  | Dominant | 0.16 | 1.93 (0.77-4.85) |
|  |  |  |  |  | Recessive | 0.52 | 0.69 (0.20-2.45) |
|  |  |  |  |  |  |  |  |
| rs17875394 | G/G | A/G | A/A | 0.73 | Heterozygous | 1.00 | 0.88 (0.26-3.04) |
| Cases | 68 | 12 | 2 |  | Homozygous | 1.00 | NA |
| Controls | 20 | 4 | 0 |  | Dominant | 1.00 | 1.03 (0.31-3.48) |
|  |  |  |  |  | Recessive | 1.00 | NA |
|  |  |  |  |  |  |  |  |
| rs1632943 | A/A | A/C | C/C | 0.21 | Heterozygous | 0.08 | 2.44 (0.89-6.74) |
| Cases | 27 | 44 | 11 |  | Homozygous | 1.00 | 1.22 (0.32-4.63) |
| Controls | 12 | 8 | 4 |  | Dominant | 0.13 | 2.04 (0.81-5.13) |
|  |  |  |  |  | Recessive | 0.74 | 0.78 (0.22-2.70) |
|  |  |  |  |  |  |  |  |
| rs1233333 | A/A | A/G | G/G | 0.21 | Heterozygous | 0.09 | 2.36 (0.86-6.49) |
| Cases | 28 | 44 | 10 |  | Homozygous | 1.00 | 1.07 (0.28-4.10) |
| Controls | 12 | 8 | 4 |  | Dominant | 0.16 | 1.93 (0.77-4.85) |
|  |  |  |  |  | Recessive | 0.52 | 0.69 (0.20-2.45) |
|  |  |  |  |  |  |  |  |
| Any form of GVHD |  |  |  |  |  |  |  |
| rs1736936 | A/A | A/G | G/G | 0.33 | Heterozygous | 0.14 | 2.04 (0.78-5.36) |
| Cases | 50 | 68 | 19 |  | Homozygous | 1.00 | 1.14 (0.33-3.97) |
| Controls | 12 | 8 | 4 |  | Dominant | 0.21 | 1.74 (0.73-4.16) |
|  |  |  |  |  | Recessive | 0.75 | 0.81 (0.25-2.61) |
|  |  |  |  |  |  |  |  |
| rs1736935 | G/G | A/G | A/A | 0.30 | Heterozygous | 0.13 | 2.07 (0.79-5.44) |
| Cases | 50 | 69 | 18 |  | Homozygous | 1.00 | 1.08 (0.31-3.78) |
| Controls | 12 | 8 | 4 |  | Dominant | 0.21 | 1.74 (0.73-4.16) |
|  |  |  |  |  | Recessive | 0.75 | 0.76 (0.23-2.47) |
|  |  |  |  |  |  |  |  |
| rs3823321 | G/G | A/G | A/A | 0.01* | Heterozygous | 0.99 | 1.01 (0.35-2.88) |
| Cases | 69 | 54 | 14 |  | Homozygous | 0.01* | 0.23 (0.08-0.69) |
| Controls | 9 | 7 | 8 |  | Dominant | 0.25 | 0.59 (0.24-1.44) |
|  |  |  |  |  | Recessive | 0.01* | 0.23 (0.08-0.63) |
|  |  |  |  |  |  |  |  |
| rs1736934 | A/A | A/T | T/T | 0.19 | Heterozygous | 0.11 | 2.14 (0.82-5.57) |
| Cases | 64 | 60 | 13 |  | Homozygous | 0.45 | 3.25 (0.40-26.71) |
| Controls | 16 | 7 | 1 |  | Dominant | 0.07 | 2.28 (0.92-5.68) |
|  |  |  |  |  | Recessive | 0.70 | 2.41 (0.30-19.34) |
|  |  |  |  |  |  |  |  |
| rs1632947 | A/A | A/G | G/G | 0.33 | Heterozygous | 0.14 | 2.04 (0.78-5.36) |
| Cases | 50 | 68 | 19 |  | Homozygous | 1.00 | 1.14 (0.33-3.97) |
| Controls | 12 | 8 | 4 |  | Dominant | 0.21 | 1.74 (0.73-4.16) |
|  |  |  |  |  | Recessive | 0.75 | 0.81 (0.25-2.61) |
|  |  |  |  |  |  |  |  |
| rs1632946 | T/T | C/T | C/C | 0.33 | Heterozygous | 0.14 | 2.04 (0.78-5.36) |
| Cases | 50 | 68 | 19 |  | Homozygous | 1.00 | 1.14 (0.33-3.97) |
| Controls | 12 | 8 | 4 |  | Dominant | 0.21 | 1.74 (0.73-4.16) |
|  |  |  |  |  | Recessive | 0.75 | 0.81 (0.25-2.61) |
|  |  |  |  |  |  |  |  |
| rs1233334 | C/C | C/G | C/T | 0.34 | Heterozygous | 0.28 | 0.17 (0.01-2.82) |
| Cases | 135 | 1 | 1 |  | Heterozygous | 1.00 | NA |
| Controls | 23 | 1 | 0 |  |  |  |  |
|  |  |  |  |  |  |  |  |
| rs2249863 | G/G | T/G | T/T | 0.33 | Heterozygous | 0.14 | 2.04 (0.78-5.36) |
| Cases | 50 | 68 | 19 |  | Homozygous | 1.00 | 1.14 (0.33-3.97) |
| Controls | 12 | 8 | 4 |  | Dominant | 0.21 | 1.74 (0.73-4.16) |
|  |  |  |  |  | Recessive | 0.75 | 0.81 (0.25-2.61) |
|  |  |  |  |  |  |  |  |
| rs2735022 | G/G | A/G | A/A | 0.33 | Heterozygous | 0.14 | 2.04 (0.78-5.36) |
| Cases | 50 | 68 | 19 |  | Homozygous | 1.00 | 1.14 (0.33-3.97) |
| Controls | 12 | 8 | 4 |  | Dominant | 0.21 | 1.74 (0.73-4.16) |
|  |  |  |  |  | Recessive | 0.75 | 0.81 (0.25-2.61) |
|  |  |  |  |  |  |  |  |
| rs35674592 | T/T | T/G | G/G | 0.33 | Heterozygous | 0.14 | 2.04 (0.78-5.36) |
| Cases | 50 | 68 | 19 |  | Homozygous | 1.00 | 1.14 (0.33-3.97) |
| Controls | 12 | 8 | 4 |  | Dominant | 0.21 | 1.74 (0.73-4.16) |
|  |  |  |  |  | Recessive | 0.75 | 0.81 (0.25-2.61) |
|  |  |  |  |  |  |  |  |
| rs1632944 | A/A | A/G | G/G | 0.33 | Heterozygous | 0.14 | 2.04 (0.78-5.36) |
| Cases | 50 | 68 | 19 |  | Homozygous | 1.00 | 1.14 (0.33-3.97) |
| Controls | 12 | 8 | 4 |  | Dominant | 0.21 | 1.74 (0.73-4.16) |
|  |  |  |  |  | Recessive | 0.75 | 0.81 (0.25-2.62) |
|  |  |  |  |  |  |  |  |
| rs1736933 | C/C | A/C | A/A | 0.32 | Heterozygous | 0.13 | 2.08 (0.79-5.48) |
| Cases | 49 | 68 | 20 |  | Homozygous | 1.00 | 1.22 (0.35-4.25) |
| Controls | 12 | 8 | 4 |  | Dominant | 0.19 | 1.80 (0.75-4.30) |
|  |  |  |  |  | Recessive | 0.76 | 0.86 (0.26-2.76) |
|  |  |  |  |  |  |  |  |
| rs1736932 | G/G | C/G | C/C | 0.31 | Heterozygous | 0.15 | 2.03 (0.77-5.33) |
| Cases | 51 | 69 | 17 |  | Homozygous | 1.00 | 1.00 (0.28-3.52) |
| Controls | 12 | 8 | 4 |  | Dominant | 0.24 | 1.24 (0.71-4.03) |
|  |  |  |  |  | Recessive | 0.52 | 0.52 (0.43-4.63) |
|  |  |  |  |  |  |  |  |
| rs17875394 | G/G | A/G | A/A | 0.70 | Heterozygous | 1.00 | 1.05 (0.33-3.35) |
| Cases | 110 | 23 | 4 |  | Homozygous | 1.00 | NA |
| Controls | 20 | 4 | 0 |  | Dominant | 1.00 | 1.23 (0.39-3.89) |
|  |  |  |  |  | Recessive | 1.00 | NA |
|  |  |  |  |  |  |  |  |
| rs1632943 | A/A | A/C | C/C | 0.30 | Heterozygous | 0.12 | 2.11 (0.80-5.55) |
| Cases | 49 | 69 | 19 |  | Homozygous | 1.00 | 1.16 (0.33-4.06) |
| Controls | 12 | 8 | 4 |  | Dominant | 0.19 | 1.80 (0.75-4.30) |
|  |  |  |  |  | Recessive | 0.75 | 0.81 (0.25-2.61) |
|  |  |  |  |  |  |  |  |
| rs1233333 | A/A | A/G | G/G | 0.33 | Heterozygous | 0.14 | 2.04 (0.78-5.36) |
| Cases | 50 | 68 | 19 |  | Homozygous | 1.00 | 1.14 (0.33-3.97) |
| Controls | 12 | 8 | 4 |  | Dominant | 0.21 | 1.74 (0.73-4.16) |
|  |  |  |  |  | Recessive | 0.75 | 0.81 (0.25-2.61) |
| ^a^Cases: patients with the event; Controls: patients are alive, without relapse, or without GVHD; *:p < 0.05; NA: not applicable. | | | | | | | |

**Supplementary Table S3.** Association study of the haplotypes in 3’-UTR of HLA-G with various adverse outcomes post-HSCT.

| **Haplotype** | **No. of patients (%)** | | | | **Additive p** | **Model** | **Logistic regression p** | **OR (95 % CI)** |
| --- | --- | --- | --- | --- | --- | --- | --- | --- |
|  | **2** | **1** | | **0** |  |  |  |  |
| Mortality |  | |  |  |  |  |  |  |
| UTR-1 |  | |  |  |  |  |  |  |
| Cases | 9 | | 35 | 27 | 0.92 | Heterozygous (2 vs 1) | 0.87 | 1.09 (0.41-2.87) |
| Controls | 12 | | 43 | 38 |  | Homozygous (2 vs 0) | 0.92 | 0.95 (0.35-2.56) |
|  |  | |  |  |  |  |  |  |
| UTR-2 |  | |  |  |  |  |  |  |
| Cases | 0 | | 13 | 58 | 0.58 | Heterozygous (2 vs 1) | 1.00 | NA |
| Controls | 0 | | 14 | 79 |  | Homozygous (2 vs 0) | 0.58 | 0.79 (0.35-1.81) |
|  |  | |  |  |  |  |  |  |
| UTR-3 |  | |  |  |  |  |  |  |
| Cases | 6 | | 31 | 34 | 0.22 | Heterozygous (2 vs 1) | 0.09 | 2.50 (0.86-7.29) |
| Controls | 15 | | 31 | 47 |  | Homozygous (2 vs 0) | 0.27 | 1.81 (0.64-5.14) |
|  |  | |  |  |  |  |  |  |
| UTR-4 |  | |  |  |  |  |  |  |
| Cases | 0 | | 1 | 70 | 1.00 | Heterozygous (2 vs 1) | 1.00 | NA |
| Controls | 0 | | 1 | 92 |  | Homozygous (2 vs 0) | 1.00 | 0.76 (0.05-12.38) |
|  |  | |  |  |  |  |  |  |
| UTR-5 |  | |  |  |  |  |  |  |
| Cases | 0 | | 0 | 71 | 0.51 | Heterozygous (2 vs 1) | 1.00 | NA |
| Controls | 0 | | 2 | 91 |  | Homozygous (2 vs 0) | 0.51 | NA |
|  |  | |  |  |  |  |  |  |
| UTR-7 |  | |  |  |  |  |  |  |
| Cases | 2 | | 28 | 41 | 0.51 | Heterozygous (2 vs 1) | 0.44 | 2.33 (0.42-13.02) |
| Controls | 5 | | 30 | 58 |  | Homozygous (2 vs 0) | 0.70 | 1.77 (0.33-9.56) |
|  |  | |  |  |  |  |  |  |
| Relapse |  | |  |  |  |  |  |  |
| UTR-1 |  | |  |  |  |  |  |  |
| Cases | 6 | | 38 | 23 | 0.13 | Heterozygous (2 vs 1) | 0.10 | 0.42 (0.15-1.20) |
| Controls | 15 | | 40 | 42 |  | Homozygous (2 vs 0) | 0.57 | 0.73 (0.25-2.14) |
|  |  | |  |  |  |  |  |  |
| UTR-2 |  | |  |  |  |  |  |  |
| Cases | 0 | | 9 | 58 | 0.38 | Heterozygous (2 vs 1) | 1.00 | NA |
| Controls | 0 | | 18 | 79 |  | Homozygous (2 vs 0) | 0.38 | 0.68 (0.29-1.62) |
|  |  | |  |  |  |  |  |  |
| UTR-3 |  | |  |  |  |  |  |  |
| Cases | 8 | | 29 | 30 | 0.49 | Heterozygous (2 vs 1) | 0.49 | 0.70 (0.25-1.93) |
| Controls | 13 | | 33 | 51 |  | Homozygous (2 vs 0) | 0.93 | 1.05 (0.39-2.81) |
|  |  | |  |  |  |  |  |  |
| UTR-4 |  | |  |  |  |  |  |  |
| Cases | 0 | | 0 | 67 | 0.51 | Heterozygous (2 vs 1) | 1.00 | NA |
| Controls | 0 | | 2 | 95 |  | Homozygous (2 vs 0) | 0.51 | N/A |
|  |  | |  |  |  |  |  |  |
| UTR-5 |  | |  |  |  |  |  |  |
| Cases | 0 | | 1 | 66 | 1.00 | Heterozygous (2 vs 1) | 1.00 | NA |
| Controls | 0 | | 1 | 96 |  | Homozygous (2 vs 0) | 1.00 | 1.46 (0.09-23.67) |
|  |  | |  |  |  |  |  |  |
| UTR-7 |  | |  |  |  |  |  |  |
| Cases | 1 | | 27 | 39 | 0.23 | Heterozygous (2 vs 1) | 0.13 | 0.19 (0.02-1.69) |
| Controls | 6 | | 31 | 60 |  | Homozygous (2 vs 0) | 0.25 | 0.26 (0.03-2.21) |
|  |  | |  |  |  |  |  |  |
| Grades I–II |  | |  |  |  |  |  |  |
| UTR-1 |  | |  |  |  |  |  |  |
| Cases | 19 | | 21 | 6 | 0.41 | Heterozygous (2 vs 1) | 0.43 | 2.00 (0.43-9.21) |
| Controls | 13 | | 7 | 4 |  | Homozygous (2 vs 0) | 1.00 | 0.97 (0.23-4.15) |
|  |  | |  |  |  |  |  |  |
| UTR-2 |  | |  |  |  |  |  |  |
| Cases | 0 | | 3 | 43 | 1.00 | Heterozygous (2 vs 1) | 1.00 | NA |
| Controls | 0 | | 2 | 22 |  | Homozygous (2 vs 0) | 1.00 | 1.30 (0.20-8.38) |
|  |  | |  |  |  |  |  |  |
| UTR-3 |  | |  |  |  |  |  |  |
| Cases | 7 | | 20 | 19 | 0.19 | Heterozygous (2 vs 1) | 0.08 | 3.27 (0.86-12.35) |
| Controls | 8 | | 7 | 9 |  | Homozygous (2 vs 0) | 0.18 | 2.41 (0.67-8.74) |
|  |  | |  |  |  |  |  |  |
| UTR-4 |  | |  |  |  |  |  |  |
| Cases | 0 | | 0 | 46 | 0.34 | Heterozygous (2 vs 1) | 1.00 | NA. |
| Controls | 0 | | 1 | 23 |  | Homozygous (2 vs 0) | 0.34 | NA |
|  |  | |  |  |  |  |  |  |
| UTR-5 |  | |  |  |  |  |  |  |
| Cases | 0 | | 2 | 44 | 0.54 | Heterozygous (2 vs 1) | 1.00 | NA |
| Controls | 0 | | 0 | 24 |  | Homozygous (2 vs 0) | 0.54 | NA |
|  |  | |  |  |  |  |  |  |
| UTR-7 |  | |  |  |  |  |  |  |
| Cases | 2 | | 15 | 29 | 0.58 | Heterozygous (2 vs 1) | 1.00 | 1.50 (0.11-20.30) |
| Controls | 1 | | 5 | 18 |  | Homozygous (2 vs 0) | 1.00 | 0.81 (0.07-9.54) |
|  |  | |  |  |  |  |  |  |
| Grades III–IV |  | |  |  |  |  |  |  |
| UTR-1 |  | |  |  |  |  |  |  |
| Cases | 4 | | 3 | 2 | 0.87 | Heterozygous (2 vs 1) | 1.00 | 0.86 (0.10-7.51) |
| Controls | 13 | | 7 | 4 |  | Homozygous (2 vs 0) | 0.63 | 0.62 (0.08-4.70) |
|  |  | |  |  |  |  |  |  |
| UTR-2 |  | |  |  |  |  |  |  |
| Cases | 0 | | 1 | 8 | 1.00 | Heterozygous (2 vs 1) | 1.00 | NA |
| Controls | 0 | | 2 | 22 |  | Homozygous (2 vs 0) | 1.00 | 0.73 (0.06-9.16) |
|  |  | |  |  |  |  |  |  |
| UTR-3 |  | |  |  |  |  |  |  |
| Cases | 0 | | 5 | 4 | 0.12 | Heterozygous (2 vs 1) | 0.06 | NA |
| Controls | 8 | | 7 | 9 |  | Homozygous (2 vs 0) | 0.13 | NA |
|  |  | |  |  |  |  |  |  |
| UTR-4 |  | |  |  |  |  |  |  |
| Cases | 0 | | 0 | 9 | 1.00 | Heterozygous (2 vs 1) | 1.00 | NA |
| Controls | 0 | | 1 | 23 |  | Homozygous (2 vs 0) | 1.00 | NA |
|  |  | |  |  |  |  |  |  |
| UTR-5 |  | |  |  |  |  |  |  |
| Cases | 0 | | 0 | 9 | NA | Heterozygous (2 vs 1) | 1.00 | NA |
| Controls | 0 | | 0 | 24 |  | Homozygous (2 vs 0) | NA | NA |
|  |  | |  |  |  |  |  |  |
| UTR-7 |  | |  |  |  |  |  |  |
| Cases | 1 | | 3 | 5 | 0.52 | Heterozygous (2 vs 1) | 1.00 | 0.60 (0.03-13.58) |
| Controls | 1 | | 5 | 18 |  | Homozygous (2 vs 0) | 0.43 | 0.28 (0.02-5.27) |
|  |  | |  |  |  |  |  |  |
| Chronic GVHD |  | |  |  |  |  |  |  |
| UTR-1 |  | |  |  |  |  |  |  |
| Cases | 29 | | 47 | 9 | 0.08 | Heterozygous (2 vs 1) | 0.20 | 2.98 (0.72-12.35) |
| Controls | 13 | | 7 | 4 |  | Homozygous (2 vs 0) | 1.00 | 0.99 (0.26-3.81) |
|  |  | |  |  |  |  |  |  |
| UTR-2 |  | |  |  |  |  |  |  |
| Cases | 0 | | 21 | 64 | 0.08 | Heterozygous (2 vs 1) | 1.00 | NA |
| Controls | 0 | | 2 | 22 |  | Homozygous (2 vs 0) | 0.08 | 0.28 (0.06-1.28) |
|  |  | |  |  |  |  |  |  |
| UTR-3 |  | |  |  |  |  |  |  |
| Cases | 6 | | 30 | 49 | <0.01* | Heterozygous (2 vs 1) | 0.01* | 5.71 (1.50-21.84) |
| Controls | 8 | | 7 | 9 |  | Homozygous (2 vs 0) | <0.01* | 7.26 (2.03-25.98) |
|  |  | |  |  |  |  |  |  |
| UTR-4 |  | |  |  |  |  |  |  |
| Cases | 0 | | 1 | 84 | 0.39 | Heterozygous (2 vs 1) | 1.00 | NA |
| Controls | 0 | | 1 | 23 |  | Homozygous (2 vs 0) | 0.39 | 3.65 (0.22-60.66) |
|  |  | |  |  |  |  |  |  |
| UTR-5 |  | |  |  |  |  |  |  |
| Cases | 0 | | 0 | 85 | NA | Heterozygous (2 vs 1) | 1.00 | NA |
| Controls | 0 | | 0 | 24 |  | Homozygous (2 vs 0) | NA | NA |
|  |  | |  |  |  |  |  |  |
| UTR-7 |  | |  |  |  |  |  |  |
| Cases | 3 | | 35 | 47 | 0.19 | Heterozygous (2 vs 1) | 0.46 | 2.33 (0.20-27.03) |
| Controls | 1 | | 5 | 18 |  | Homozygous (2 vs 0) | 1.00 | 0.87 (0.09-8.92) |
|  |  | |  |  |  |  |  |  |
| Any form of GVHD |  | |  |  |  |  |  |  |
| UTR-1 |  | |  |  |  |  |  |  |
| Cases | 52 | | 71 | 17 | 0.15 | Heterozygous (2 vs 1) | 0.24 | 2.39 (0.63-9.09) |
| Controls | 13 | | 7 | 4 |  | Homozygous (2 vs 0) | 1.00 | 0.94 (0.27-3.28) |
|  |  | |  |  |  |  |  |  |
| UTR-2 |  | |  |  |  |  |  |  |
| Cases | 0 | | 25 | 115 | 0.37 | Heterozygous (2 vs 1) | 1.00 | NA |
| Controls | 0 | | 2 | 22 |  | Homozygous (2 vs 0) | 0.37 | 0.42 (0.10-1.89) |
|  |  | |  |  |  |  |  |  |
| UTR-3 |  | |  |  |  |  |  |  |
| Cases | 13 | | 55 | 72 | 0.01* | Heterozygous (2 vs 1) | 0.02* | 4.84 (1.49-15.75) |
| Controls | 8 | | 7 | 9 |  | Homozygous (2 vs 0) | 0.01* | 4.92 (1.61-15.10) |
|  |  | |  |  |  |  |  |  |
| UTR-4 |  | |  |  |  |  |  |  |
| Cases | 0 | | 1 | 139 | 0.27 | Heterozygous (2 vs 1) | 1.00 | NA |
| Controls | 0 | | 1 | 23 |  | Homozygous (2 vs 0) | 0.27 | 6.04 (0.37-100.05) |
|  |  | |  |  |  |  |  |  |
| UTR-5 |  | |  |  |  |  |  |  |
| Cases | 0 | | 2 | 138 | 1.00 | Heterozygous (2 vs 1) | 1.00 | NA |
| Controls | 0 | | 0 | 24 |  | Homozygous (2 vs 0) | 1.00 | NA |
|  |  | |  |  |  |  |  |  |
| UTR-7 |  | |  |  |  |  |  |  |
| Cases | 6 | | 53 | 81 | 0.26 | Heterozygous (2 vs 1) | 0.51 | 1.77 (0.18-17.75) |
| Controls | 1 | | 5 | 18 |  | Homozygous (2 vs 0) | 1.00 | 0.75 (0.09-6.62) |
| Dose of haplotype: 2 means that the indicated haplotype is present in both strands of DNA. 1 means that only one strand of DNA carries the indicated haplotypes, and the other strand carries the other haplotype. 0 means that the indicated haplotype is absence in both strands of DNA.  Cases: patients with the event; Controls: patients are alive, without relapse, or without GVHD. | | | | | | | | |

**Supplementary Table S4.** Association study of the haplotypes in 5’-URR of HLA-G with various adverse outcomes post-HSCT.

| **Haplotype** | **No. of patients (%)** | | | **Additive p** | **Model** | **Logistic regression p** | **OR (95 % CI)** |
| --- | --- | --- | --- | --- | --- | --- | --- |
|  | **2** | **1** | **0** |  |  |  |  |
| Mortality |  |  |  |  |  |  |  |
| 010101a |  |  |  |  |  |  |  |
| Cases | 9 | 33 | 26 | 0.98 | Heterozygous (2 vs 1) | 1.00 | 1.00 (0.38-2.65) |
| Controls | 12 | 44 | 37 |  | Homozygous (2 vs 0) | 0.90 | 0.94 (0.35-2.55) |
|  |  |  |  |  |  |  |  |
| 010101b |  |  |  |  |  |  |  |
| Cases | 0 | 1 | 67 | 1.00 | Heterozygous (2 vs 1) | 1.00 | NA |
| Controls | 0 | 1 | 92 |  | Homozygous (2 vs 0) | 1.00 | 0.73 (0.05-11.85) |
|  |  |  |  |  |  |  |  |
| 010102a |  |  |  |  |  |  |  |
| Cases | 6 | 28 | 34 | 0.10 | Heterozygous (2 vs 1) | 0.94 | 0.96 (0.30-3.07) |
| Controls | 8 | 39 | 46 |  | Homozygous (2 vs 0) | 0.98 | 0.99 (0.31-3.11) |
|  |  |  |  |  |  |  |  |
| 0103a |  |  |  |  |  |  |  |
| Cases | 0 | 0 | 68 | 1.00 | Heterozygous (2 vs 1) | 1.00 | NA |
| Controls | 0 | 1 | 92 |  | Homozygous (2 vs 0) | 1.00 | NA |
|  |  |  |  |  |  |  |  |
| 0104a |  |  |  |  |  |  |  |
| Cases | 3 | 23 | 42 | 0.26 | Heterozygous (2 vs 1) | 0.12 | 3.00 (0.72-12.52) |
| Controls | 9 | 23 | 61 |  | Homozygous (2 vs 0) | 0.36 | 2.07 (0.53-8.08) |
|  |  |  |  |  |  |  |  |
| 0104b |  |  |  |  |  |  |  |
| Cases | 1 | 13 | 54 | 0.64 | Heterozygous (2 vs 1) | 0.61 | 2.79 (0.26-30.27) |
| Controls | 3 | 14 | 76 |  | Homozygous (2 vs 0) | 0.64 | 2.13 (0.22-21.05) |
|  |  |  |  |  |  |  |  |
| Relapse |  |  |  |  |  |  |  |
| 010101a |  |  |  |  |  |  |  |
| Cases | 6 | 38 | 22 | 0.10 | Heterozygous (2 vs 1) | 0.09 | 0.41 (0.14-1.17) |
| Controls | 15 | 39 | 41 |  | Homozygous (2 vs 0) | 0.59 | 0.75 (0.25-2.19) |
|  |  |  |  |  |  |  |  |
| 010101b |  |  |  |  |  |  |  |
| Cases | 0 | 0 | 66 | 0.51 | Heterozygous (2 vs 1) | 1.00 | NA |
| Controls | 0 | 2 | 93 |  | Homozygous (2 vs 0) | 0.51 | NA |
|  |  |  |  |  |  |  |  |
| 010102a |  |  |  |  |  |  |  |
| Cases | 3 | 33 | 30 | 0.11 | Heterozygous (2 vs 1) | 0.06 | 0.28 (0.07-1.10) |
| Controls | 11 | 34 | 50 |  | Homozygous (2 vs 0) | 0.37 | 0.46 (0.12-1.76) |
|  |  |  |  |  |  |  |  |
| 0103a |  |  |  |  |  |  |  |
| Cases | 0 | 0 | 66 | 1.00 | Heterozygous (2 vs 1) | 1.00 | NA |
| Controls | 0 | 1 | 94 |  | Homozygous (2 vs 0) | 1.00 | NA |
|  |  |  |  |  |  |  |  |
| 0104a |  |  |  |  |  |  |  |
| Cases | 3 | 21 | 42 | 0.43 | Heterozygous (2 vs 1) | 0.32 | 0.40 (0.10-1.66) |
| Controls | 9 | 25 | 61 |  | Homozygous (2 vs 0) | 0.36 | 0.48 (0.12-1.90) |
|  |  |  |  |  |  |  |  |
| 0104b |  |  |  |  |  |  |  |
| Cases | 1 | 14 | 51 | 0.39 | Heterozygous (2 vs 1) | 0.60 | 0.31 (0.03-3.36) |
| Controls | 3 | 13 | 79 |  | Homozygous (2 vs 0) | 1.00 | 0.52 (0.05-5.10) |
|  |  |  |  |  |  |  |  |
| Chronic GVHD |  |  |  |  |  |  |  |
| 010101a |  |  |  |  |  |  |  |
| Cases | 9 | 45 | 28 | 0.09 | Heterozygous (2 vs 1) | 0.21 | 2.86 (0.69-11.85) |
| Controls | 4 | 7 | 13 |  | Homozygous (2 vs 0) | 1.00 | 0.96 (0.25-3.69) |
|  |  |  |  |  |  |  |  |
| 010101b |  |  |  |  |  |  |  |
| Cases | 0 | 1 | 81 | 0.40 | Heterozygous (2 vs 1) | 1.00 | NA |
| Controls | 0 | 1 | 23 |  | Homozygous (2 vs 0) | 0.40 | 3.52 (0.21-58.51) |
|  |  |  |  |  |  |  |  |
| 010102a |  |  |  |  |  |  |  |
| Cases | 9 | 40 | 33 | 0.07 | Heterozygous (2 vs 1) | 1.00 | 0.64 (0.07-5.83) |
| Controls | 1 | 7 | 16 |  | Homozygous (2 vs 0) | 0.25 | 0.23 (0.03-1.97) |
|  |  |  |  |  |  |  |  |
| 0103a |  |  |  |  |  |  |  |
| Cases | 0 | 0 | 82 | NA | Heterozygous (2 vs 1) | 1.00 | NA |
| Controls | 0 | 0 | 24 |  | Homozygous (2 vs 0) | NA | NA |
|  |  |  |  |  |  |  |  |
| 0104a |  |  |  |  |  |  |  |
| Cases | 2 | 22 | 58 | <0.01* | Heterozygous (2 vs 1) | 0.01* | 9.43 (1.54-57.74) |
| Controls | 6 | 7 | 11 |  | Homozygous (2 vs 0) | <0.01* | 15.82 (2.82-88.80) |
|  |  |  |  |  |  |  |  |
| 0104b |  |  |  |  |  |  |  |
| Cases | 2 | 12 | 68 | 0.73 | Heterozygous (2 vs 1) | 1.00 | NA |
| Controls | 0 | 4 | 20 |  | Homozygous (2 vs 0) | 1.00 | NA |
|  |  |  |  |  |  |  |  |
| Grades I–II |  |  |  |  |  |  |  |
| 010101a |  |  |  |  |  |  |  |
| Cases | 6 | 22 | 18 | 0.32 | Heterozygous (2 vs 1) | 0.42 | 2.10 (0.46-9.623 |
| Controls | 4 | 7 | 13 |  | Homozygous (2 vs 0) | 1.00 | 0.92 (0.22-3.95) |
|  |  |  |  |  |  |  |  |
| 010101b |  |  |  |  |  |  |  |
| Cases | 0 | 0 | 46 | 0.34 | Heterozygous (2 vs 1) | 1.00 | NA |
| Controls | 0 | 1 | 23 |  | Homozygous (2 vs 0) | 0.34 | NA |
|  |  |  |  |  |  |  |  |
| 010102a |  |  |  |  |  |  |  |
| Cases | 3 | 17 | 26 | 0.70 | Heterozygous (2 vs 1) | 1.00 | 0.81 (0.08-9.18) |
| Controls | 1 | 7 | 16 |  | Homozygous (2 vs 0) | 1.00 | 0.54 (0.05-5.66) |
|  |  |  |  |  |  |  |  |
| 0103a |  |  |  |  |  |  |  |
| Cases | 0 | 1 | 45 | 1.00 | Heterozygous (2 vs 1) | 1.00 | NA |
| Controls | 0 | 0 | 24 |  | Homozygous (2 vs 0) | 1.00 | NA |
|  |  |  |  |  |  |  |  |
| 0104a |  |  |  |  |  |  |  |
| Cases | 3 | 15 | 28 | 0.09 | Heterozygous (2 vs 1) | 0.11 | 4.29 (0.82-22.34) |
| Controls | 6 | 7 | 11 |  | Homozygous (2 vs 0) | 0.05 | 5.09 (1.08-24.02) |
|  |  |  |  |  |  |  |  |
| 0104b |  |  |  |  |  |  |  |
| Cases | 2 | 9 | 35 | 0.54 | Heterozygous (2 vs 1) | 1.00 | NA |
| Controls | 0 | 4 | 20 |  | Homozygous (2 vs 0) | 0.54 | NA |
|  |  |  |  |  |  |  |  |
| Grades III–IV |  |  |  |  |  |  |  |
| 010101a |  |  |  |  |  |  |  |
| Cases | 2 | 3 | 4 | 0.87 | Heterozygous (2 vs 1) | 1.00 | 0.86 (0.10-7.51) |
| Controls | 4 | 7 | 13 |  | Homozygous (2 vs 0) | 0.63 | 0.62 (0.09-4.70) |
|  |  |  |  |  |  |  |  |
| 010101b |  |  |  |  |  |  |  |
| Cases | 0 | 0 | 9 | 1.00 | Heterozygous (2 vs 1) | 1.00 | NA |
| Controls | 0 | 1 | 23 |  | Homozygous (2 vs 0) | 1.00 | NA |
|  |  |  |  |  |  |  |  |
| 010102a |  |  |  |  |  |  |  |
| Cases | 1 | 3 | 5 | 0.71 | Heterozygous (2 vs 1) | 1.00 | 0.43 (0.02-9.36) |
| Controls | 1 | 7 | 16 |  | Homozygous (2 vs 0) | 0.46 | 0.31 (0.02-5.96) |
|  |  |  |  |  |  |  |  |
| 0103a |  |  |  |  |  |  |  |
| Cases | 0 | 0 | 9 | NA | Heterozygous (2 vs 1) | 1.00 | NA |
| Controls | 0 | 0 | 24 |  | Homozygous (2 vs 0) | NA | NA |
|  |  |  |  |  |  |  |  |
| 0104a |  |  |  |  |  |  |  |
| Cases | 1 | 2 | 6 | 0.53 | Heterozygous (2 vs 1) | 1.00 | 1.71 (0.12-23.94) |
| Controls | 6 | 7 | 11 |  | Homozygous (2 vs 0) | 0.63 | 3.27 (0.32-33.94) |
|  |  |  |  |  |  |  |  |
| 0104b |  |  |  |  |  |  |  |
| Cases | 0 | 2 | 7 | 1.00 | Heterozygous (2 vs 1) | 1.00 | NA |
| Controls | 0 | 4 | 20 |  | Homozygous (2 vs 0) | 1.00 | 0.70 (0.10-4.70) |
|  |  |  |  |  |  |  |  |
| Any form of GVHD |  |  |  |  |  |  |  |
| 010101a |  |  |  |  |  |  |  |
| Cases | 17 | 70 | 50 | 0.14 | Heterozygous (2 vs 1) | 0.24 | 2.35 (0.62-8.97) |
| Controls | 4 | 7 | 13 |  | Homozygous (2 vs 0) | 1.00 | 0.91 (0.26-3.15) |
|  |  |  |  |  |  |  |  |
| 010101b |  |  |  |  |  |  |  |
| Cases | 0 | 1 | 136 | 0.28 | Heterozygous (2 vs 1) | 1.00 | NA |
| Controls | 0 | 1 | 23 |  | Homozygous (2 vs 0) | 0.28 | 5.91 (0.36-97.90) |
|  |  |  |  |  |  |  |  |
| 010102a |  |  |  |  |  |  |  |
| Cases | 13 | 60 | 64 | 0.19 | Heterozygous (2 vs 1) | 1.00 | 0.66 (0.08-5.83) |
| Controls | 1 | 7 | 16 |  | Homozygous (2 vs 0) | 0.45 | 0.31 (0.04-2.53) |
|  |  |  |  |  |  |  |  |
| 0103a |  |  |  |  |  |  |  |
| Cases | 0 | 4 | 136 | 1.00 | Heterozygous (2 vs 1) | 1.00 | NA |
| Controls | 0 | 0 | 24 |  | Homozygous (2 vs 0) | 1.00 | NA |
|  |  |  |  |  |  |  |  |
| 0104a |  |  |  |  |  |  |  |
| Cases | 6 | 39 | 92 | <0.01* | Heterozygous (2 vs 1) | 0.01* | 5.57 (1.39-22.33) |
| Controls | 6 | 7 | 11 |  | Homozygous (2 vs 0) | <0.01* | 8.36 (2.30-30.47) |
|  |  |  |  |  |  |  |  |
| 0104b |  |  |  |  |  |  |  |
| Cases | 4 | 23 | 110 | 0.70 | Heterozygous (2 vs 1) | 1.00 | NA |
| Controls | 0 | 4 | 20 |  | Homozygous (2 vs 0) | 1.00 | NA |
| Dose of haplotype: 2 means that the indicated haplotype is present in both strands of DNA. 1 means that only one strand of DNA carries the indicated haplotypes, and the other strand carries the other haplotype. 0 means that the indicated haplotype is absence in both strands of DNA.  Cases: patients with the event; Controls: patients are alive, without relapse, or without GVHD. | | | | | | | |

**Supplementary Table S5.** The accession numbers for the SNPs that

are investigated in this study.

| SNP | NCBI dbSNP database accession numbers |
| --- | --- |
| rs1736936 | 2137544421 |
| rs1736935 | 5981611086 |
| rs3823321 | 5981611087 |
| rs1736934 | 5981611088 |
| rs17875389 | 5981611089 |
| rs3115630 | 5981611090 |
| rs1632947 | 5981611091 |
| rs1632946 | 5981611092 |
| rs1233334 | 5981611093 |
| rs2249863 | 5981611094 |
| rs2735022 | 5981611095 |
| rs35674592 | 5981611096 |
| rs17875391 | 5981611097 |
| rs1632944 | 5981611098 |
| rs201221694 | 5981611099 |
| rs112940953 | 5981611100 |
| rs17875393 | 5981611101 |
| rs1736933 | 5981611102 |
| rs149890776 | 5981611103 |
| rs1736932 | 5981611104 |
| rs17875394 | 5981611105 |
| rs17875395 | 5981611106 |
| rs17875396 | 5981611107 |
| rs1632943 | 5981611108 |
| rs191630481 | 5981611109 |
| rs1233333 | 5981611110 |
| rs17875397 | 5981611111 |
| rs371194629 | 5981611112 |
| rs1707 | 5981611113 |
| rs1710 | 5981611114 |
| rs17179101 | 5981611115 |
| rs17179108 | 5981611116 |
| rs1063320 | 5981611117 |
| rs9380142 | 5981611118 |
| rs1610696 | 5981611119 |
| rs1233331 | 5981611120 |
